# Fatty acid synthase inhibition improves hypertension-induced erectile dysfunction by suppressing oxidative stress and NLRP3 inflammasome-dependent pyroptosis through activating the Nrf2/HO-1 pathway

**DOI:** 10.3389/fimmu.2024.1532021

**Published:** 2025-01-14

**Authors:** Jiaochen Luan, Mengchi Yu, Qi Gu, Xuan Zhou, Yunqiang Shao, Tong Chen, Jiayi Zhang, Zheng Zhu, Ninghong Song, Jie Yang

**Affiliations:** ^1^ Department of Urology, Jiangsu Provincial People’s Hospital, The First Affiliated Hospital of Nanjing Medical University, Nanjing, China; ^2^ Department of Urology, Children's Hospital of Nanjing Medical University, Nanjing, China; ^3^ Department of Urology, People’s Hospital of Xinjiang Kizilsu Kirgiz Autonomous Prefecture, Kizilsu Kirgiz Autonomous Prefecture, China

**Keywords:** erectile dysfunction, Fasn, Nrf2/HO-1, pyroptosis, hypertension

## Abstract

**Background:**

Erectile dysfunction (ED) is a prevalent male sexual disorder, commonly associated with hypertension, though the underlying mechanisms remain poorly understood.

**Objective:**

This study aims to explore the role of Fatty acid synthase (Fasn) in hypertension-induced ED and evaluate the therapeutic potential of the Fasn inhibitor C75.

**Materials and methods:**

Erectile function was assessed by determining the intracavernous pressure/mean arterial pressure (ICP/MAP) ratio, followed by the collection of cavernous tissue for transcriptomic and non-targeted metabolomic analyses. *In vitro*, a concentration of 10^-6^ M angiotensin II (Ang II) was applied to rat aortic endothelial cells (RAOECs) to establish a model of hypertension. *In vivo*, spontaneously hypertensive rats (SHR) were randomly divided into two groups. The SHR+C75 group received intraperitoneal injections of C75 at a dose of 2 mg/kg once a week. After five weeks of treatment, the erectile function of the rats was assessed, and penile tissues were harvested for further analysis. Molecular and protein expression were assessed using Western blotting, qRT-PCR, immunofluorescence staining, and immunohistochemistry.

**Results:**

The SHR exhibited ED, indicated by reduced maximum ICP/MAP ratios. Histologically, corpus cavernosum tissue of SHR showed elevated fibrosis and endothelial dysfunction. Additionally, increased expression of the NLRP3 inflammasome, Caspase-1, GSDMD, and the pro-inflammatory cytokines IL-1β and IL-18 was observed. Multi-omics analysis revealed significant enrichment in lipid metabolic pathways, with Fasn identified as a hub gene. *In vitro*, siFasn and C75 enhanced antioxidant markers Nrf2 and HO-1, reduced ROS accumulation, and suppressed NLRP3 and GSDMD levels. *In vivo*, C75 treatment restored endothelial function and reversed erectile dysfunction, accompanied by decreased oxidative stress and pyroptosis in the penile corpus cavernosum.

**Conclusion:**

These findings suggest that Fasn inhibition may offer a promising therapeutic strategy for hypertension-induced ED by alleviating oxidative stress and suppressing NLRP3 inflammasome-dependent endothelial cell pyroptosis *via* activation of the Nrf2/HO-1 pathway.

## 1 Introduction

Erectile dysfunction (ED) is a common male sexual disorder, characterized by the persistent inability to achieve or maintain an erection sufficient for satisfactory sexual intercourse for at least three months (1). ED is estimated to affect 150 million men globally (2). The pathophysiology of ED is complex, involving oxidative stress, inflammatory responses, vascular changes, and alterations in vasoactive substances (3). As penile erection relies on vascular events, including venous occlusion, various diseases that induce vascular changes, such as diabetes mellitus, metabolic syndrome, and hyperlipidemia, are linked to ED (4). Notably, hypertension is associated with an increased prevalence of ED, affecting up to 68% of individuals, which is 2-3 times higher than the general population (5, 6). Elevated blood pressure can severely damage the vascular system, increasing the risk of heart attacks, strokes, and chronic renal disease (7, 8). Previous studies have shown that the penile corpus cavernosum in spontaneously hypertensive rats (SHR) exhibits alterations in smooth muscle, endothelium, and extracellular matrix (9, 10). At the same time, dysfunctional β-adrenergic-mediated contraction and endothelium-dependent relaxation were observed in the penile corpus cavernosum in SHR, which may be related to the overproduction of cyclooxygenase products (11, 12). However, the exact mechanisms by which hypertension induces ED remain incompletely understood.

Emerging evidence highlights the critical role of lipid metabolism dysregulation in the development and pathophysiology of hypertension (13, 14). Short-chain fatty acids have been implicated in blood pressure regulation (15). Cao et al. demonstrated that alterations in the gut microbiome and lipid metabolism contribute to left ventricular hypertrophy in hypertensive individuals (16). Animal studies have shown that SHR have significantly elevated levels of plasma triglycerides and free fatty acids compared to Wistar-Kyoto (WKY) rats (17). Furthermore, research has established a non-linear positive correlation between the Metabolic Score for Visceral Fat and ED incidence (18). Despite these findings, the role of lipid metabolism in hypertension-induced ED remains underexplored. Fatty acid synthase (Fasn), encoded by the Fasn gene, is a key enzyme involved in the synthesis of long-chain fatty acids (19). While Fasn expression is typically low or absent in normal cells, it is upregulated under specific pathological conditions, including tumors, inflammatory diseases, and cardiovascular disorders (20–23).

Oxidative stress is characterized by excessive reactive oxygen species (ROS) production and an imbalance in antioxidant defenses (24). ROS are recognized as intracellular signaling molecules with cytotoxic effects and play a central role in oxidative stress (25). The production of ROS is closely linked to the onset of numerous diseases, including ED. Elevated oxidative stress in the penis of diabetic rat models is believed to contribute to ED progression (26). Maintaining ROS homeostasis through the antioxidant system is essential for physiological functions, such as cell signaling, cellular function, and metabolic regulation (27). Nrf2, a key transcription factor in the antioxidant stress response, regulates antioxidant genes, including HO-1, by binding to AREs (28). Pyroptosis is a form of programmed cell death associated with inflammation (29). This process, triggered by inflammasome activation, is marked by cell swelling, membrane rupture, and the release of intracellular contents, leading to a pronounced inflammatory response (30). The NLRP3 inflammasome activates pro-Caspase-1, which is subsequently processed into its active form. Caspase-1 promotes the maturation of IL-1β and IL-18, while also cleaving GSDMD into GSDMD-C and GSDMD-N (31). During pyroptosis, GSDMD-N inserts into the cell membrane, forming cytotoxic pores that compromise membrane integrity and facilitate the release of inflammatory mediators (32). Exogenous stimuli, such as LPS, can induce ROS production, activate the NLRP3 inflammasome, and trigger Caspase-1-dependent pyroptosis in cardiomyocytes (33). Research has shown that ROS generation plays a role in NLRP3 inflammasome-mediated pyroptosis, contributing to the pathogenesis of various inflammatory diseases, including heart failure (34), metabolic syndrome (34), diabetes (35), and liver fibrosis (36). Emerging evidence increasingly supports a role for pyroptosis in the progression of ED. Chen et al. demonstrated that a low androgenic state impairs erectile function by inducing pyroptosis (29). Further studies have shown that either the knockdown of NLRP3 or the administration of NLRP3 inhibitors can ameliorate erectile function by mitigating pyroptosis, particularly in diabetic rats with ED (37, 38).

Overall, we hypothesize that oxidative stress and NLRP3 inflammasome-dependent pyroptosis are critical in the pathogenesis of hypertension-induced ED. Using non-targeted metabolomics and RNA sequencing, this study investigates the mechanisms underlying hypertension-induced ED. Lipid metabolism and Fasn were identified as central players in this process. Knockdown of Fasn or treatment with C75, a Fasn inhibitor, reduced ROS levels and pyroptosis by activating the Nrf2/HO-1 pathway, ultimately improving endothelial dysfunction and reversing hypertension-induced ED. The therapeutic potential of C75 offers novel insights and a theoretical basis for treating hypertension-induced ED.

## 2 Methods

### 2.1 Animal procedures

The experiment utilized 10-week-old WKY rats (n = 20) as the control group (Norm group) and 10-week-old SHR (n = 20), sourced from Vital River Laboratory Animal Technology Co., Ltd. (Beijing, China). The study was approved by the Institutional Animal Care and Use Committee of Nanjing Medical University (Ethics Review Number: IACUC-2207013). All animals were acclimatized to a controlled environment (temperature: 22 ± 2°C; 12-hour light-dark cycle) for at least one week prior to the experiment. In the rescue experiment, the SHR+C75 group received intraperitoneal injections of the Fasn inhibitor C75 (Sigma-Aldrich, USA) at a dose of 2 mg/kg once a week for 5 weeks, dissolved in 8% DMSO. The SHR group was administered an equivalent volume of 8% DMSO *via* the same route (39).

### 2.2 Erectile evaluation

For physiological assessments, rats were anesthetized with intraperitoneal pentobarbital sodium (40 mg/kg). The carotid artery was exposed and cannulated to measure mean arterial pressure (MAP). Concurrently, a heparinized 25-gauge needle was inserted into the corpus cavernosum to record intracavernous pressure (ICP). The cavernous nerve was dissected from the outer edge of the prostate, and electrical stimulation was applied using a bipolar electrode (5V, 15 Hz, 0.2 ms pulse width) for 1 minute. The BL-420 S biological function system (Chengdu Taimeng Technology Co., Ltd., China) was used for pressure measurement and electrical stimulation generation. This procedure was repeated three times to ensure accuracy. Erectile function was evaluated by calculating the maximum ICP/MAP ratio. At the end of the experiment, rats were euthanized, and the corpus cavernosum tissue was collected.

### 2.3 Masson trichrome staining

The midshaft penile tissue was preserved in 4% paraformaldehyde overnight, then dehydrated, embedded in paraffin, and sectioned to a thickness of 5 µm. Masson’s trichrome staining was performed according to standard protocols, and the smooth muscle-to-collagen ratio was analyzed using ImageJ software (version 1.54d).

### 2.4 Immunofluorescence assay

Fluorescence microscopy was employed to assess the expression of target proteins in paraffin-embedded corpus cavernosum tissues and rat aortic endothelial cell (RAOECs). Sections were incubated overnight with primary antibodies at 4°C. The primary antibodies were as follows: anti-α-SMA (Proteintech, 14395-1-ap, 1:800), anti-VWF (Proteintech, 27186-1-ap, 1:100), anti-CD31 (Proteintech, 66065-2-Ig, 1:800), anti-eNOS (Cell signaling technology, 32027S, 1:200), anti-Nrf2 (Proteintech, 16396-1-ap, 1:100), anti-Fasn (ABclonal, A0461, 1:100), anti-HO-1 (ABclonal, A1346, 1:100), anti-NLRP3 (ABclonal, A5652, 1:100) and anti-NQO1(Abcam, ab80588, 1:50). After washing, they were incubated with the appropriate secondary antibodies for 1 hour. The secondary antibodies included goat anti-rabbit Alexa Fluor 488 (Abcam, Ab150077, 1:500), goat anti-rabbit Alexa Fluor 594 (Abcam, Ab150088, 1:500), and goat anti-mouse Alexa Fluor 594 (Abcam, Ab150116, 1:500). Nuclei were stained with DAPI (Beyotime, China). Images were captured using a Zeiss LSM 5 Live confocal microscope (Zeiss, Oberkochen, Germany) and analyzed using ImageJ software.

### 2.5 Immunohistochemistry

Corpus cavernosum tissues were fixed in 4% paraformaldehyde at 4°C and embedded in paraffin. Sections (5 µm thick) were prepared for subsequent experiments. Sections were incubated with the primary antibodies overnight at 4°C. The primary antibodies included anti-collagen I (Origene, TA309096, 1:100), anti-NLRP3 (ABclonal, A5652, 1:100), anti-Caspase-1 (Affinity, AF5418, 1:50), anti-IL-18(Abmart, M027287, 1:100), anti-IL-1β (Servicebio, GB11113, 1:800), anti-Caspase 9 (Proteintech, 10380-1-ap, 1:100), anti-PPAR-γ (Proteintech, 16643-1-ap, 1:400), anti-Caspase 3 (Abmart, T40044, 1:200), anti-Fasn (ABclonal, A0461, 1:100), anti-Scd1(ABclonal, A16429, 1:100), anti-Acaca (ABclonal, A15606, 1:100), anti-Dgat2 (ABclonal, A13891, 1:100) and anti-Acsl1(ABclonal, A16253, 1:100). After being washed with PBS, the sections underwent incubation with a rabbit/mouse HRP-conjugated secondary antibody (TPB-0015, Typing) and were subsequently exposed to DAB to make the reaction sites visible. Images were captured using an upright microscope.

### 2.6 Measurement of nitric oxide, and cGMP

Penile corpora cavernosa tissues were homogenized in lysis buffer, and the supernatants were collected for cGMP and NO detection. cGMP was measured using the rat cGMP ELISA kit (JM-01434R2, JINGMEI), while NO was quantified using the nitrate-nitrite assay kit (A012-1-2, Nanjing Jiancheng), following the standard protocol.

### 2.7 Western blotting analysis

For protein extraction from tissues and cells, RIPA lysis buffer (Beyotime, Shanghai, China) supplemented with a protease and phosphatase inhibitor cocktail (Beyotime) was used. Protein samples were separated by SDS-PAGE and transferred to PVDF membranes. After blocking with 5% non-fat milk, membranes were incubated overnight at 4°C with a specific primary antibody. The primary antibodies included anti-GAPDH (Cell signaling technology, 5174S, 1:1000), anti-eNOS (Cell signaling technology, 32027S, 1:1000), anti-Fasn (ABclonal, A0461, 1:1000), anti-VWF (Proteintech, 11778-1-ap, 1:1000), anti-β-tubulin (Proteintech, 10094-1-ap, 1:5000), anti-Nrf2 (Proteintech, 16396-1-ap, 1:2000), anti-HO-1(ABclonal, A1346, 1:2000), anti-NLRP3 (ABclonal, A5652, 1:1000), anti-Caspase-1 (Affinity, AF5418, 1:1000), anti-cleaved-Caspase-1 (Affinity, AF4005, 1:1000) and anti-GSDMD-N (Affinity, DF13758, 1:1000). Following three washes with TBST, membranes were incubated with a secondary antibody for 90 minutes. Protein bands were visualized using Bio-Rad’s Western ECL Substrate kit and quantified using ImageJ software.

### 2.8 Metabolomics analysis

Metabolite profiling was performed *via* liquid chromatography electrospray tandem mass spectrometry (LC-ESI-MS/MS). Differentially accumulated metabolites (DAMs) were identified based on VIP values > 1 and P-values < 0.05. Metabolic pathway analysis was conducted using the KEGG Compound Database. Detailed methods for non-targeted metabolomics analyses are provided in the **Supplementary Methods.**

### 2.9 Transcriptome analysis

RNA extraction from the outer urethra membrane of penile cavernous tissue was performed using a TRIzol reagent. cDNA libraries were subsequently constructed and sequenced using the Illumina platform. Differentially expressed genes (DEGs) were identified and functionally analyzed. Detailed methods for transcriptome analysis are provided in the **Supplementary Methods.**

### 2.10 qRT-PCR

Total RNA was extracted from corpus cavernosum tissue and cell lines, and cDNA was synthesized using HiScript II reagent (Vazyme, China). qRT-PCR was performed using the StepOne Plus Real-Time PCR system (Applied Biosystems, USA) to quantify transcript levels. The fold change in target mRNA expression was determined using the 2^−ΔΔCt^ method. The PCR primer sequences used in qRT-PCR are provided in **Supplementary Table S1**.

### 2.11 Cell treatment and Fasn knockdown *in vitro*

RAOECs were purchased from Procell Life Science & Technology Co. Cells were cultured in high-glucose DMEM supplemented with 10% fetal bovine serum and maintained at 37°C with 5% CO2. A 10^-6^ M concentration of AngII (MedChemExpress, USA) was used to induce an *in vitro* hypertensive environment. Fasn knockdown in RAOECs was achieved using small interfering RNA (siRNA) designed by GeneRay Biotechnology (Shanghai, China). Cells were transfected with siRNA and transfection reagents, cultured for 24 hours, then transferred to a fresh medium for an additional 24 hours. The Fasn siRNA sequences are listed in **Supplementary Table S2**. The ROS inhibitor (NAC) was obtained from Beyotime (Shanghai, China).

### 2.12 Detection of ROS generation

The dihydroethidium (DHE) probe was employed to assess intracellular ROS levels in RAOECs. After removing the medium, 1 mL of 10 μM DHE (APPLYGEN, China) was added to the cells. Following a 30-minute incubation at 37°C in the dark, the cells were washed three times with 0.01 M PBS. ROS levels were visualized using a fluorescence microscope.

### 2.13 Measurement of levels of MDA, SOD and GSH

MDA concentration was used to represent oxidative stress levels, while superoxide dismutase (SOD) and glutathione (GSH) concentrations were used to reflect antioxidant activity in penile corpora cavernosa. The levels of MDA (A003-1-1), SOD (A001-1-1) and GSH (A006-1-1) were assessed using respective measurement kits (Nanjing Jiancheng, China) according to the manufacturer’s instructions.

### 2.14 Clonogenic assays

RAOECs were seeded in 6-well plates at a density of 500 cells per well and incubated in a 37°C, 5% CO2 incubator for 14 days, with medium replacement every four days. After incubation, the cells were fixed with 4% paraformaldehyde and stained with 1% crystal violet. Colony formation was quantified using an optical microscope.

### 2.15 Cell viability assay

After transfection, RAOECs were seeded in 96-well plates. After complete cell adhesion, hypertension was simulated using AngII at various concentrations. Cell viability was evaluated 48 hours post-treatment using the Cell Counting Kit-8 (CCK-8, Vazyme, Jiangsu, China). Absorbance at 450 nm was measured using a Synergy4 microplate reader (BioTek, Winooski, VT, USA).

### 2.16 5-ethynyl-2′-deoxyuridine assay

Cell proliferation was assessed by incubating RAOECs with 100 μL of EdU solution at 37°C for 4 hours, followed by fixation with 4% paraformaldehyde. The cells were stained with 1× Apollo reaction cocktail and incubated with Hoechst 33342 for 30 minutes. Images were captured using a fluorescence microscope.

### 2.17 Terminal deoxynucleoitidyl transferase dUTP nick‐end labeling staining

Tissue apoptosis was evaluated using a TUNEL assay kit (Vazyme, China). After fixation and permeabilization, samples were incubated with the TUNEL reaction solution. Stained samples were imaged using a fluorescence microscope.

### 2.18 Flow cytometric apoptosis assay

Cell apoptosis levels were determined using the Annexin V-FITC/PI assay kit (Vazyme, Biotech). After digestion and centrifugation, cells were washed twice with PBS and resuspended in a binding buffer. Annexin V-FITC and PI staining solution were added, and cells were incubated for 10 minutes. Apoptosis was quantified using the CytoFLEX flow cytometer (Beckman).

### 2.19 Detection of the levels of IL-18 and IL-1β

After collecting the cell culture supernatants, the concentrations of inflammatory cytokines IL-18 (ERC010.96) and IL-1β (ERC007.96) were quantified using ELISA kits (NeoBioscience), and the OD values were measured at 450 nm.

### 2.20 Statistical analysis

Statistical analysis was performed using SPSS 26.0 and R 4.2.1. Data are presented as Mean ± SEM. The Student’s t-test, one-way ANOVA followed by Tukey’s *post hoc* test, and the Mann–Whitney test were employed, with statistical significance set at P < 0.05.

## 3 Results

### 3.1 Assessment of ICP_max_/MAP

**Figure 1:**
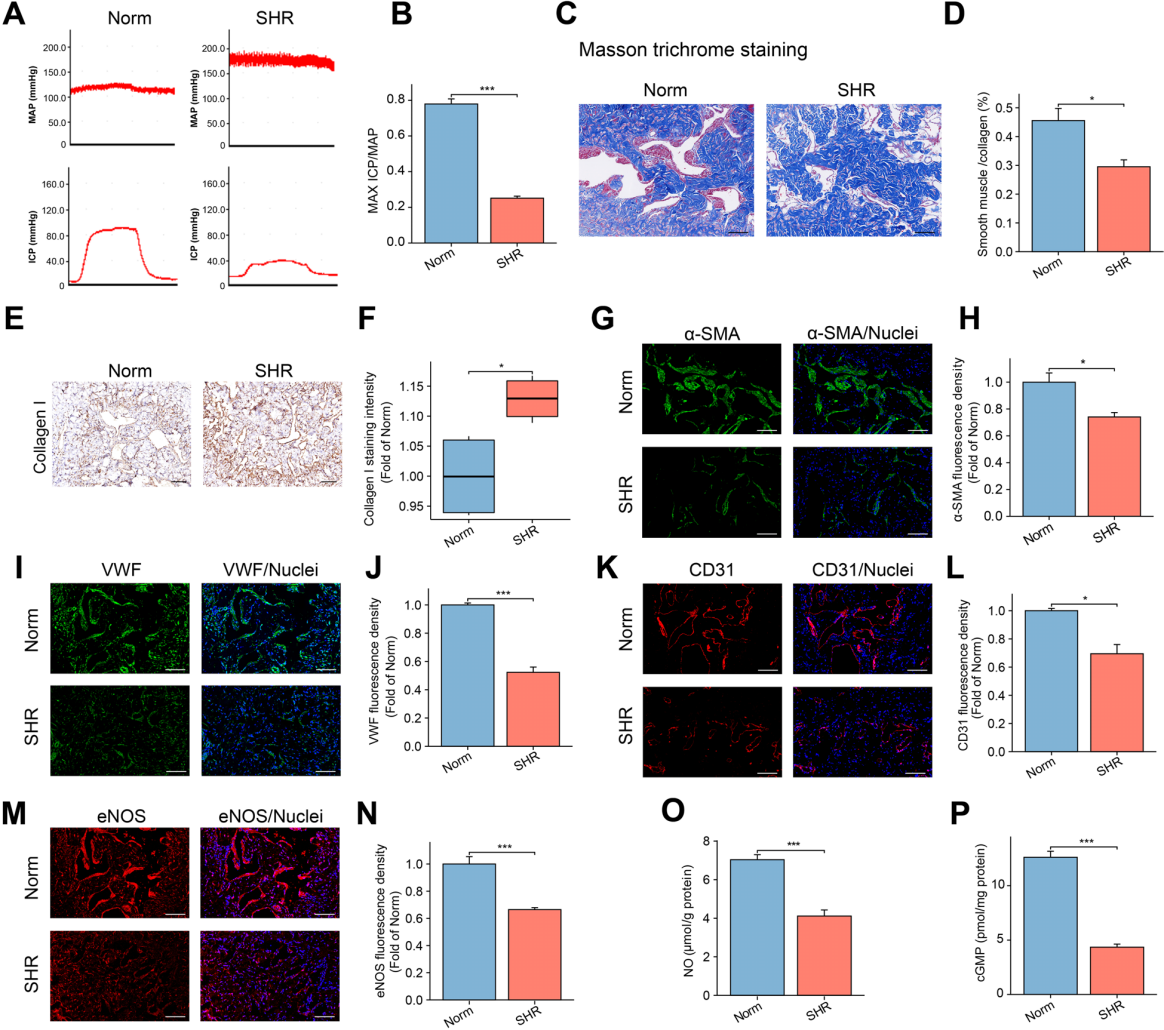
Assessment of Erectile Function in the SHR and Norm groups. **(A)** The representative images of ICP and MAP under electrical stimulation of the cavernosal nerve. **(B)** The bar graph of the maximum ICP/MAP ratio. Data are expressed as mean ± SEM (n = 5). ***P < 0.001. T test was used. **(C)** The representative images of Masson’s trichrome staining in the corpus cavernosum tissue, with smooth muscle stained red and collagen blue. Scale bar = 100 μm. **(D)** The bar graph of smooth muscle/collagen content ratio. Data are expressed as mean ± SEM (n = 4). *P < 0.05. T test was used. **(E)** The representative image of Collagen I immunohistochemical staining. Scale bar = 100 μm. **(F)** The box graph of the quantification of Collagen I staining intensity. Data are expressed as mean ± SEM (n = 4). *P < 0.05. T test was used. **(G, I, K, M)** The immunofluorescence staining images of α-SMA **(G)**, VWF **(I)**, CD31 **(K)**, and eNOS **(M)** in the corpus cavernosum. Scale bar = 100 μm. **(H, J, L, N)** The bar graph of comparison of α-SMA **(H)**, VWF **(J)**, CD31 **(L)**, and eNOS **(N)** expression between the two groups. Data are expressed as mean ± SEM (n = 4). *P < 0.05, ***P < 0.001. T test was used. **(O, P)** The bar graph of concentrations of NO **(O)** and cGMP **(P)** between the two groups. Data are expressed as mean ± SEM (n = 6). ***P < 0.001. T test was used. SHR, spontaneously hypertensive rats; Norm, normal rats; ICP, intracavernous pressure; MAP, mean arterial blood pressure.

The maximum ICP to MAP ratio was used to assess erectile function. As shown in **Figures 1A, B**, hypertension was associated with ED, as evidenced by a significantly lower maximum ICP/MAP ratio in the SHR group compared to the Norm group (P < 0.001).

### 3.2 Hypertension induces fibrosis and decreases smooth muscle content in the penis

Masson trichrome staining was employed to evaluate the severity of fibrosis in the penile corpora cavernosa by assessing the smooth muscle-to-collagen ratio. Compared to the Norm group, the SHR group displayed a disorganized corpora cavernosa structure and a significant reduction in the smooth muscle-to-collagen ratio (**Figures 1C, D**).

Immunohistochemical analysis of Collagen I (a marker of fibrosis) confirmed that hypertension led to increased collagen deposition in the SHR group (**Figures 1E, F**). Additionally, immunofluorescence staining for α-SMA revealed a notable decrease in α-SMA content in the SHR group compared to the Norm group (**Figures 1G, H**).

### 3.3 Hypertension damages endothelial function in the penis by inhibiting the eNOS/NO/cGMP pathway

To explore the impact of hypertension on endothelial cells in the penile corpora cavernosa, immunofluorescence analysis was conducted to detect endothelial cell markers (VWF, CD31, and eNOS). The expression levels of VWF, CD31, and eNOS were significantly reduced in the SHR group compared to the Norm group (**Figures 1I–N**). eNOS, a specific isoform of NOS, facilitates the synthesis of NO from L-arginine (40). A lower level of NO and cGMP was found in the SHR group compared to the Norm group (**Figures 1O, P**).

### 3.4 Increased oxidative stress and pyroptosis in the penile corpora cavernosa of SHR

**Figure 2:**
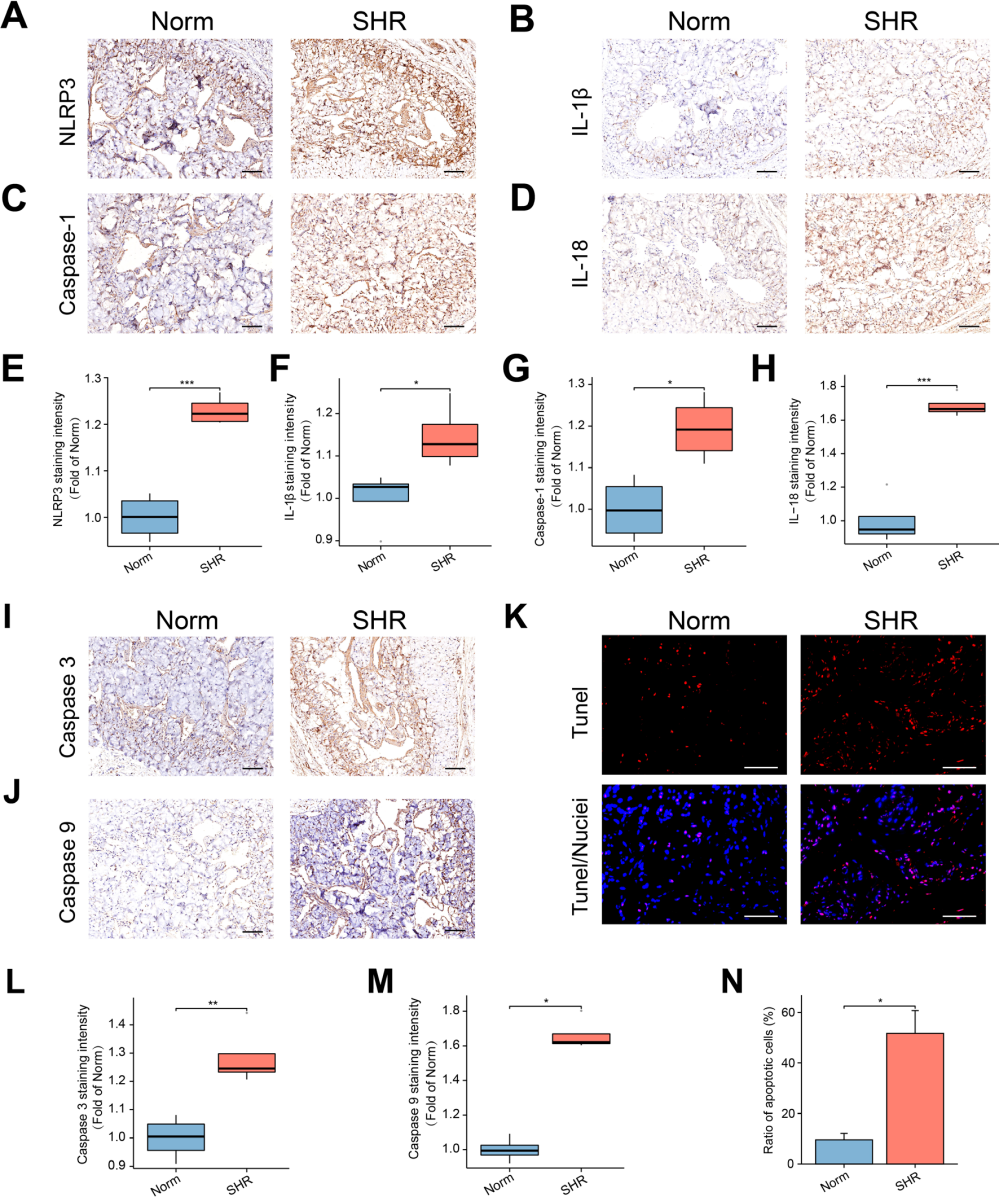
The NLRP3 inflammasome-mediated pyroptosis and apoptosis in the penile corpora cavernosa. **(A–D)** The representative images of immunohistochemical staining for NLRP3 **(A)**, IL-1β **(B)**, Caspase-1 **(C)**, and IL-18 **(D)** in the corpus cavernosum. Scale bar = 100 μm. **(E–H)** The box graphs of the quantitative data of NLRP3 **(E)**, IL-1β **(F)**, Caspase-1 **(G)**, and IL-18 **(H)** staining intensity. Data are expressed as mean ± SEM (n = 4). *P < 0.05, ***P < 0.001. T test was used. **(I, J)** The representative images of immunohistochemical staining of Caspase 3 **(I)**, and Caspase 9 **(J)** in the corpus cavernosum. Scale bars = 100 μm. **(K)** The representative images of TUNEL staining. Scale bar = 100 μm. **(L, M)** The box graphs of the quantitative data for Caspase 3 **(L)** and Caspase 9 **(M)** staining intensity. Data are expressed as mean ± SEM (n = 4). *P < 0.05, **P < 0.01. T test was used for Caspase 3, and Mann-Whitney test was used for Caspase 9. **(N)** The bar graph of the percentage of TUNEL-positive cells. Data are expressed as mean ± SEM (n = 4). *P < 0.05. T test was used.

MDA, a marker of lipid peroxidation, was used to assess oxidative stress levels, while antioxidant substances like SOD and GSH are involved in scavenging ROS. The SHR group exhibited significantly higher MDA levels and notably lower SOD and GSH levels compared to the Norm group (**Supplementary Figure S1**). To investigate inflammation and pyroptosis, immunohistochemistry was employed to assess the expression of key molecules in the penile corpora cavernosa. The expression of NLRP3 and Caspase-1 was significantly higher in the SHR group compared to the Norm group (**Figures 2A, C, E, G**). Moreover, there was a significant increase in the pro-inflammatory cytokines IL-1β and IL-18 in the corpora cavernosa of SHR (**Figures 2B, D, F, H**).

### 3.5 Hypertension induces apoptosis in the penile corpora cavernosa

Under normal conditions, Caspase 9 exists as a proenzyme and is activated during apoptotic signaling. Activated Caspase 9 can further activate Caspase 3, a critical effector enzyme in apoptosis. Immunohistochemical analysis revealed elevated expression of Caspase 3 in the SHR group, consistent with the upregulation of Caspase 9 (**Figures 2I, J, L, M**). Additionally, TUNEL analysis demonstrated a significant increase in the apoptosis index in the SHR group (**Figures 2K, N**).

### 3.6 The results of non-targeted metabolomics studies

**Figure 3:**
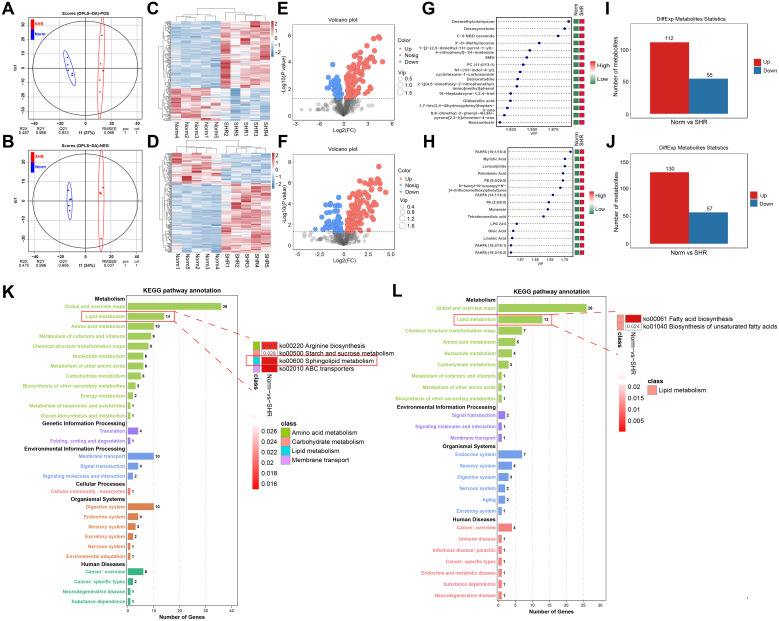
Non-targeted metabolomics studies of the corpus cavernosum. **(A, B)** The Orthogonal Partial Least Squares Discriminant Analysis (OPLS-DA) under positive and negative ionization mode. **(C, D)** The heatmaps of the differentially accumulated metabolites (DAMs) under positive and negative ionization mode. **(E, F)** The volcano plots of the DAMs under positive and negative ionization mode. **(G, H)** The top 15 ranked DAMs under positive and negative ionization mode. **(I, J)** The bar graph of the DAMs under positive and negative ionization mode. **(K, L)** The Kyoto Encyclopedia of Genes and Genomes (KEGG) pathways of the DAMs under positive and negative ionization mode.

Non-targeted metabolomics analyses of rat penile tissues from the SHR and Norm groups were conducted to explore the mechanisms underlying hypertension-induced erectile dysfunction. OPLS-DA modeling effectively separated the SHR and Norm groups in both positive and negative ionization modes (**Figures 3A, B**). Response permutation tests (RPT) confirmed model robustness, with no overfitting observed (**Supplementary Figure S2A, B**). Heatmaps of the DAMs revealed distinct differences between the two groups across both ionization modes (**Figures 3C, D**). Volcano plots demonstrated significant alterations in DAM levels due to hypertension (**Figures 3E, F**). In positive ionization mode, 167 DAMs were identified, including desmethylcitalopram, desoxycortone, C-6 NBD ceramide and 3’-O-Methylinosine, with 112 upregulated and 55 downregulated (**Figures 3G, I**; **Supplementary Table S3**). In negative ionization mode, 187 DAMs were identified, such as myristic acid, palmitoleic acid, oleic acid and linoleic acid, with 130 upregulated and 57 downregulated (**Figures 3H, J**; **Supplementary Table S4**). KEGG enrichment analysis of DAMs in both ionization modes revealed pathways related to lipid metabolism, including sphingolipid metabolism and fatty acid biosynthesis (**Figures 3K, L**). These results highlight the association between lipid metabolism and the pathophysiology of hypertension-induced ED.

### 3.7 The results of transcriptome analysis and verification of the hub genes

**Figure 4:**
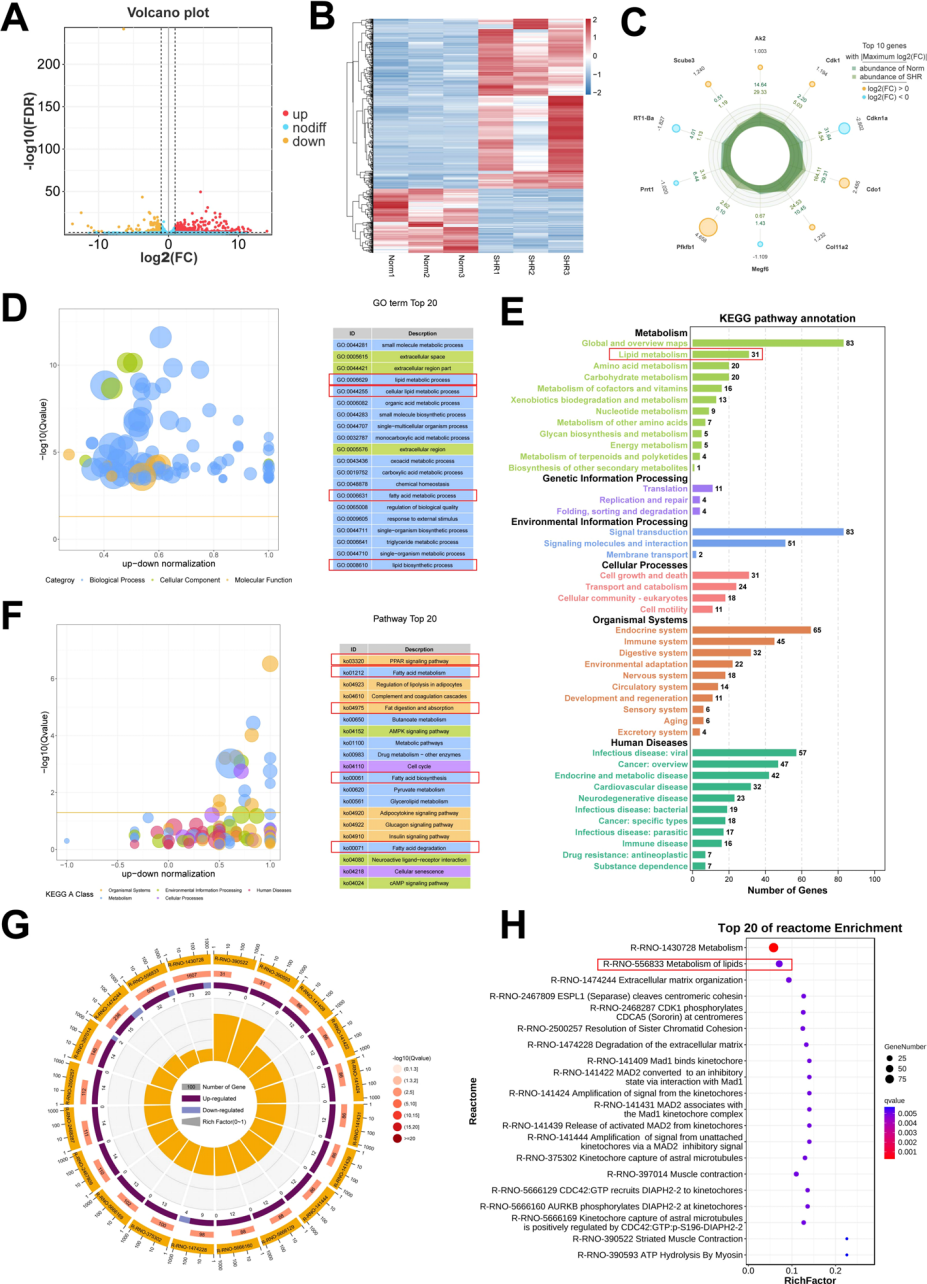
Transcriptome analysis of the corpus cavernosum. **(A)** The volcano plot of the differentially expressed genes (DEGs). **(B)** The heatmap of the DEGs. **(C)** The top 10 DEGs with Maximum |log_2_(FC)|. **(D)** Gene Ontology (GO) enrichment analysis of the DEGs. **(E, F)** Kyoto Encyclopedia of Genes and Genomes (KEGG) pathway analysis of the DEGs. **(G, H)** Reactome enrichment analysis of the DEGs.

**Figure 5:**
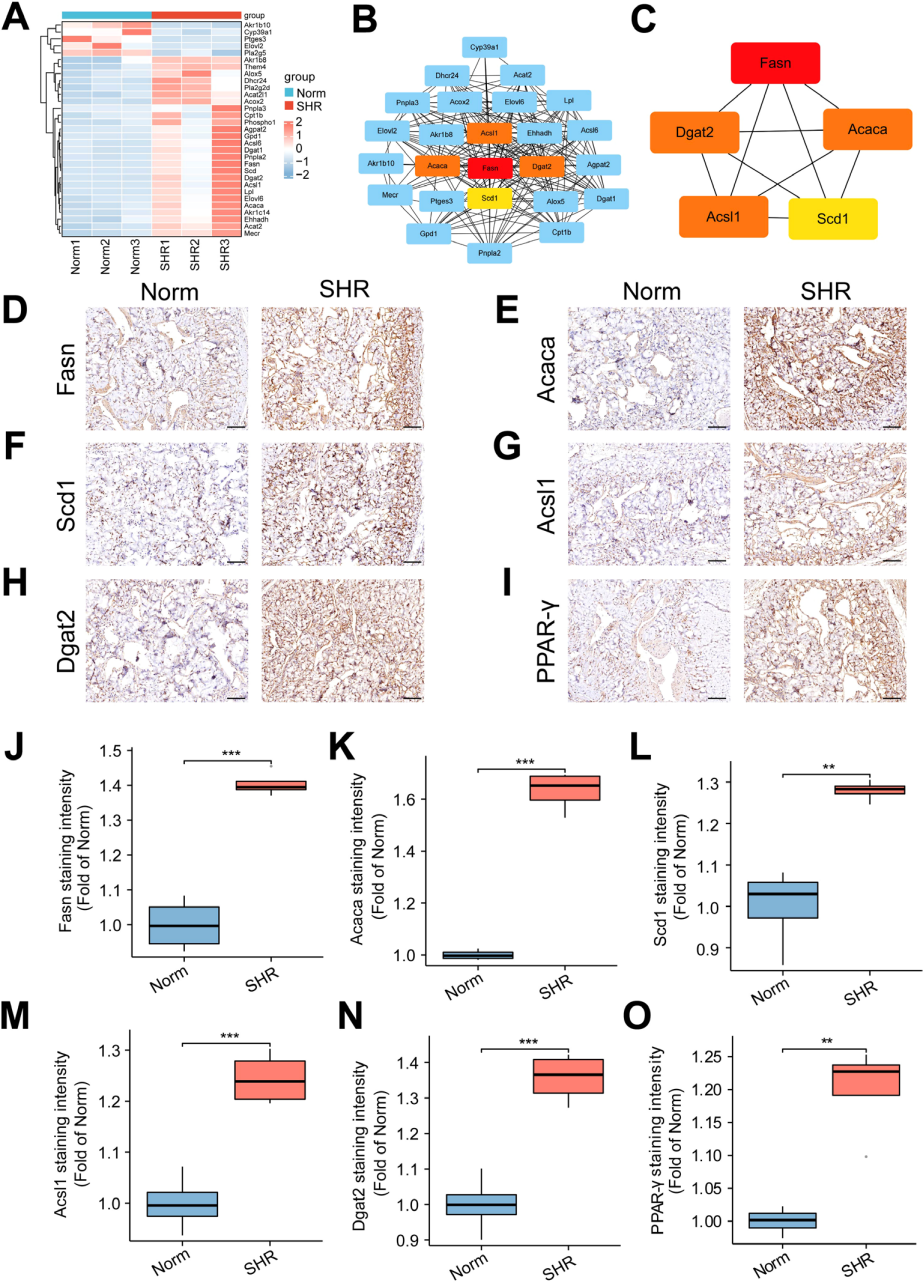
Identification and validation of lipid metabolism-related genes. **(A)** The heatmap of the differentially expressed genes (DEGs) related to lipid metabolism pathway. **(B, C)** Protein-protein interaction (PPI) networks of the DEGs constructed using STRING2 and Cytoscape software, identifying the top 5 hub genes. **(D–I)** The representative images of immunohistochemical staining for the top 5 hub genes, including Fasn **(D)**, Acaca **(E)**, Scd1 **(F)**, Acsl1 **(G)**, and Dgat2 **(H)**, as well as the core gene PPAR-γ **(I)** in the PPAR signal pathway. Scale bar = 100 μm. **(J–O)** The box graph of the quantitative data of the immunohistochemical staining for Fasn **(J)**, Acaca **(K)**, Scd1 **(L)**, Acsl1 **(M)**, Dgat2 **(N)**, and PPAR-γ **(O)**. Data are expressed as mean ± SEM (n = 4). **P < 0.01, ***P < 0.001. T test was used.

To further investigate the involvement of lipid metabolism in penile corpora cavernosa and identify potential disease targets in hypertension-induced erectile dysfunction, transcriptome analysis was performed. A total of 587 DEGs were identified between the Norm and SHR groups, including 426 upregulated and 161 downregulated genes (**Figures 4A–C**; **Supplementary Table S5**). Gene Ontology analysis revealed significant enrichment in processes related to lipid metabolism, including lipid biosynthetic process and fatty acid metabolic process (**Figure 4D**). KEGG pathway analysis highlighted significant enrichment of DEGs in fatty acid metabolism, fat digestion and absorption, fatty acid biosynthesis, and fatty acid degradation (**Figures 4E, F**). GSEA further identified enrichment in the PPAR signaling pathway and fatty acid degradation (**Supplementary Figure S3**). Reactome pathway analysis also indicated significant enrichment of DEGs in lipid metabolism (**Figures 4G, H**). These findings were consistent with the results of non-targeted metabolomics analysis. A Protein-protein interaction (PPI) network of DEGs related to lipid metabolism was constructed using STRING2 (**Figure 5A**) and analyzed through Cytoscape with cytoHubba. The top 5 genes (Fasn, Acaca, Scd1, Acsl1, and Dgat2) were identified using the Degree algorithm, with Fasn emerging as the most significant hub gene (**Figures 5B, C**).

Immunohistochemical analysis confirmed that the expression of Fasn, Acaca, Scd1, Acsl1, and Dgat2 was upregulated in the SHR group compared to the Norm group, in line with RNA-seq results. Additionally, the SHR group showed significantly higher protein expression of PPAR-γ, a key gene in the PPAR signaling pathway, compared to the Norm group (**Figures 5D–O**). These data underscore the pivotal role of lipid metabolism in hypertension-induced ED, with Fasn identified as a central gene involved in the pathogenesis.

### 3.8 Downregulation of Fasn restores the reduced cellular function in AngII-induced RAOECs

RAOECs were exposed to varying concentrations of AngII for 48 hours, and cell viability was assessed using the CCK-8 assay. The results revealed no significant impact on cell viability at AngII concentrations of 10^-8^ M and 10^-7^ M. However, at concentrations of 10^-6^ M, 10^-5^ M, and 10^-4^ M, cell viability was significantly reduced (**Supplementary Figure S4A**). Consequently, 10^-6^ M AngII was selected as the optimal concentration for further experiments.

**Figure 6:**
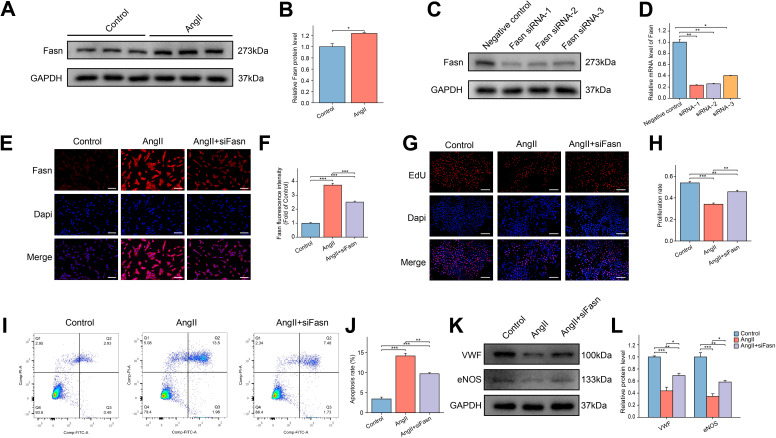
Effect of siFasn on the function of AngII induced RAOECs. **(A)** The representative WB bands of Fasn protein in the control and AngII groups in RAOECs. **(B)** The bar graph of the quantitative data of relative Fasn protein expression. Data are expressed as mean ± SEM (n = 3). *P < 0.05. T test was used. **(C)** The WB bands of Fasn protein levels after transfecting with three siRNAs that targeted Fasn. **(D)** The bar graph of the relative mRNA levels after transfecting with three siRNAs that targeted Fasn. Data are expressed as mean ± SEM (n = 3). *P < 0.05, **P < 0.01. One-way ANOVA followed by Tukey’s *post hoc* test was used. **(E)** The representative immunofluorescence images of Fasn among the control, AngII, and AngII+siFasn groups. Scale bar = 100 μm. **(F)** The bar graph of comparison of Fasn expression among the three groups. Data are expressed as mean ± SEM (n = 4). ***P < 0.001. One-way ANOVA followed by Tukey’s *post hoc* test was used. **(G)** The representative images of EdU assays among the three groups. Scale bar = 200 μm. **(H)** The bar graph of the proliferation rate among the three groups. Data are expressed as mean ± SEM (n = 4). **P < 0.01, ***P < 0.001. One-way ANOVA followed by Tukey’s *post hoc* test was used. **(I)** The representative images of flow cytometry analysis of apoptosis in RAOECs. **(J)** The flow cytometry analysis showing the apoptosis rate. Data are expressed as mean ± SEM (n = 3). **P < 0.01, ***P < 0.001. One-way ANOVA followed by Tukey’s *post hoc* test was used. **(K)** The representative WB bands showing the expression of VWF and eNOS. **(L)** Quantitative analysis of protein levels for VWF and eNOS, presented as fold change compared to the control group. Data are expressed as mean ± SEM (n = 3). *P < 0.05, **P < 0.01, ***P < 0.001. One-way ANOVA followed by Tukey’s *post hoc* test was used.

WB analysis showed a marked increase in Fasn protein expression in AngII-treated RAOECs (**Figures 6A, B**). Fasn knockdown in RAOECs was achieved using siRNA, resulting in effective inhibition of Fasn protein expression by all three siRNAs. Among them, siRNA-1 demonstrated the highest knockdown efficiency, as confirmed by both WB and PCR analysis (**Figures 6C, D**). Thus siRNA-1 was selected for subsequent functional studies. Immunofluorescence assays revealed that Fasn expression was upregulated in response to AngII treatment, but reduced in the AngII+siFasn group (**Figures 6E, F**). The EdU assay demonstrated a reduction in cell proliferation following AngII exposure, whereas the AngII+siFasn group showed enhanced proliferation compared to the AngII group (**Figures 6G, H**). Furthermore, clonogenic assays indicated that siFasn restored the decreased proliferative capacity induced by AngII (**Supplementary Figures S4B, C**). Flow cytometry analysis revealed that AngII treatment significantly increased apoptosis in RAOECs, whereas Fasn knockdown alleviated this effect (**Figures 6I, J**). Additionally, WB analysis showed a significant reduction in endothelial markers VWF and eNOS in the AngII group compared to the control group, while Fasn knockdown restored their expression (**Figures 6K, L**).

### 3.9 Downregulation of Fasn activates Nrf2/HO-1 pathway to eliminate oxidative stress and NLRP3 inflammasome-dependent pyroptosis in AngII-induced RAOECs

**Figure 7:**
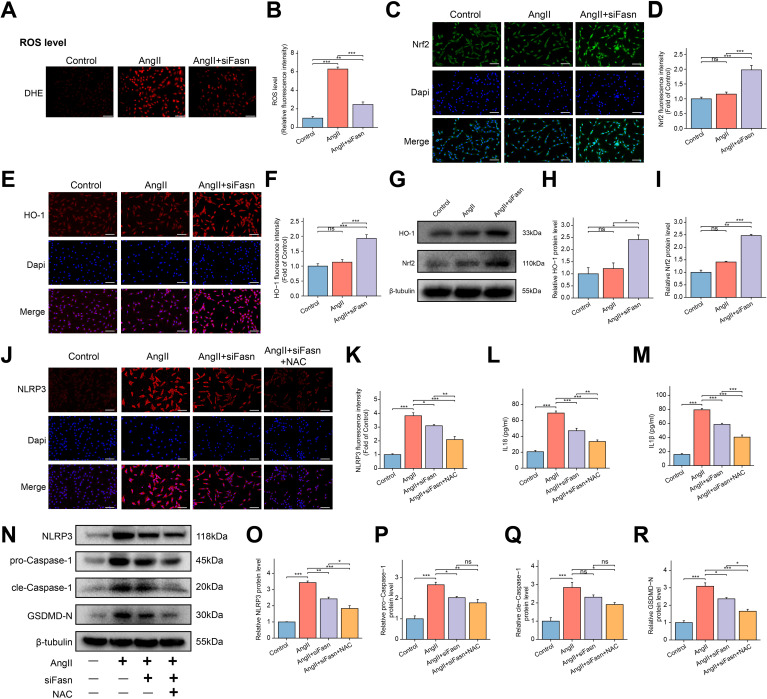
Effect of siFasn on oxidative stress and NLRP3 inflammasome-mediated pyroptosis in AngII induced RAOECs. **(A)** The representative images of dihydroethidium (DHE) staining for ROS in RAOECs. Scale bar = 200 μm. **(B)** The bar graph of quantification of ROS levels among the three groups. Data are expressed as mean ± SEM (n = 4) **P < 0.01, ***P < 0.001. One-way ANOVA followed by Tukey’s *post hoc* test was used. **(C, E)** The representative images of immunofluorescence staining of Nrf2 **(C)**, and HO-1 **(E)** in RAOECs. Scale bar = 100 μm. **(D, F)** The bar graphs of quantification of Nrf2 **(D)**, HO-1 **(F)** expression among the three groups. Data are expressed as mean ± SEM (n = 4). Ns means no significance. ***P < 0.001. One-way ANOVA followed by Tukey’s *post hoc* test was used. **(G)** The representative WB bands showing the expression of HO-1 and Nrf2. **(H, I)** Quantitative analysis of protein levels for HO-1 **(H)** and Nrf2 **(I)**, presented as fold change compared to the control group. Data are expressed as mean ± SEM (n = 3). Ns means no significance. *P < 0.05, **P < 0.01, ***P < 0.001. One-way ANOVA followed by Tukey’s *post hoc* test was used. **(J)** The representative images of immunofluorescence staining of NLRP3 in RAOECs. Scale bar = 100 μm. **(K)** The bar graphs of quantification of the fluorescence intensity for NLRP3. Data are expressed as mean ± SEM (n = 4). *P < 0.05, **P < 0.01, ***P < 0.001. One-way ANOVA followed by Tukey’s *post hoc* test was used. **(L, M)** The bar graph of the concentrations of IL-18 **(L)** and IL-1β **(M)** in the cell supernatant. Data are expressed as mean ± SEM (n = 4). **P < 0.01, ***P < 0.001. One-way ANOVA followed by Tukey’s *post hoc* test was used. **(N)** The representative WB bands showing the expression of NLRP3, pro-Caspase-1, cle-Caspase-1 and GSDMD-N protein. **(O–R)** Quantitative analysis of protein levels for NLRP3 **(O)**, pro-Caspase-1 **(P)**, cle-Caspase-1 **(Q)** and GSDMD-N **(R)**, presented as fold change compared to the control group. Data are expressed as mean ± SEM (n = 3). Ns means no significance. *P < 0.05, **P < 0.01, ***P < 0.001. One-way ANOVA followed by Tukey’s *post hoc* test was used.

To investigate whether siFasn ameliorated AngII-induced cellular dysfunction in RAOECs by exerting antioxidant effects, oxidative stress-related indicators were measured. The results indicated that AngII treatment markedly increased ROS accumulation, but siFasn treatment significantly reduced ROS levels compared to the AngII group (**Figures 7A, B**). The Nrf2/HO-1 pathway plays a critical role in cellular defense against oxidative stress (41). Immunofluorescence analysis showed no significant change in Nrf2 and HO-1 expression following AngII treatment. However, Fasn knockdown resulted in a significant increase in both Nrf2 and HO-1 expression (**Figures 7C–F**), which was corroborated by WB analysis (**Figures 7G–I**).

Immunofluorescence analysis further revealed a significant increase in NLRP3 expression in the AngII group, which was markedly reversed by Fasn knockdown (**Figures 7J, K**). Elevated levels of IL-18 and IL-1β following AngII treatment were reduced after siRNA intervention (**Figures 7L, M**). WB analysis demonstrated that Fasn knockdown reduced the expression of NLRP3, GSDMD-N, and pro-Caspase-1, which were upregulated by AngII (**Figures 7N–R**). Additionally, NAC treatment further decreased the expression of IL-18, IL-1β, NLRP3, and GSDMD-N compared to the AngII+siFasn group. However, there were no significant changes in cleaved-Caspase-1 and pro-Caspase-1 expression between the two groups (**Figures 7L, R**).

### 3.10 C75 mitigates oxidative stress and pyroptosis by activating the Nrf2/HO-1 pathway in AngII-induced RAOECs

**Figure 8:**
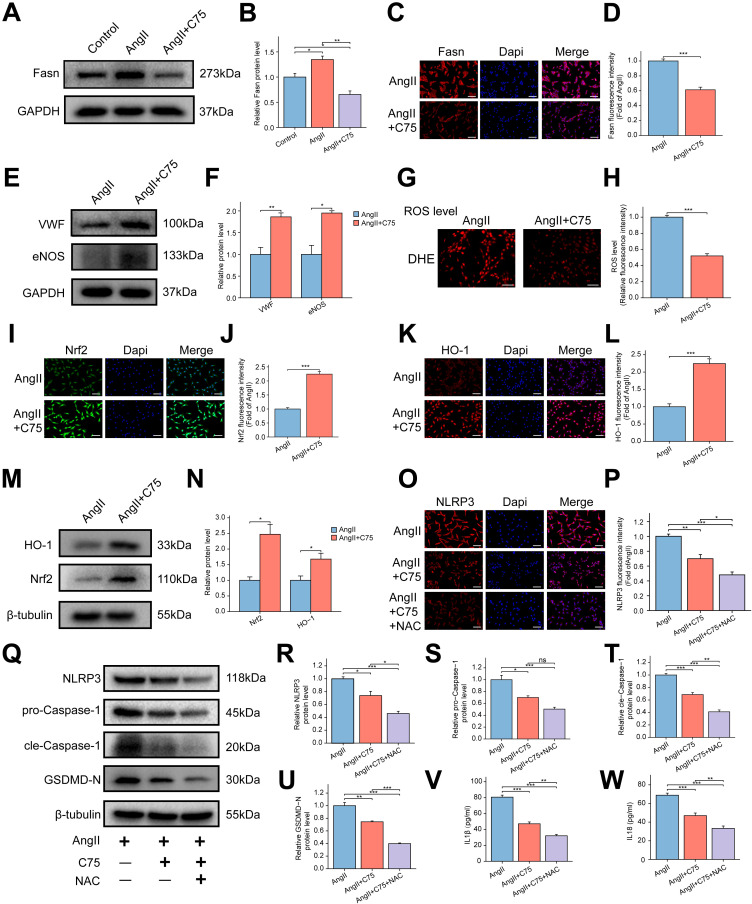
Effect of Fasn inhibitor C75 on oxidative stress and NLRP3 inflammasome-mediated pyroptosis via the Nrf2/HO-1 pathway in AngII induced RAOECs. **(A)** The representative WB bands of Fasn among the control, AngII, and AngII+C75 groups. **(B)** The bar graph of the quantitative data of Fasn protein expression. Data are expressed as mean ± SEM (n = 3). *P < 0.05, **P < 0.01. One-way ANOVA followed by Tukey’s *post hoc* test was used. **(C)** The immunofluorescence staining of Fasn between the AngII and AngII+C75 groups. **(D)** The bar graph of the quantitative data of fluorescence intensity for Fasn. Data are expressed as mean ± SEM (n = 4). ***P < 0.001. T test was used. **(E)** The representative WB bands of VWF and eNOS between the two groups. T test was used. **(F)** Quantitative analysis of protein levels for VWF and eNOS, presented as fold change compared to the control group. Data are expressed as mean ± SEM (n = 3). *P < 0.05, **P < 0.01. T test was used. **(G)** The representative images of dihydroethidium (DHE) staining for ROS between the AngII and AngII+C75 groups. Scale bar = 200 μm. **(H)** The bar graph of quantification of ROS levels. Data are expressed as mean ± SEM (n = 4). ***P < 0.001. T test was used. **(I, K)** The representative immunofluorescence images of Nrf2 **(I)** and HO-1 **(K)**. Scale bar = 100 μm. **(J, L)** The bar graph of the quantification of fluorescence intensity for Nrf2 **(J)** and HO-1 **(L)**. Data are expressed as mean ± SEM (n = 4). ***P < 0.001. T test was used. **(M)** The representative WB bands of Nrf2 and HO-1 between the two groups. **(N)** The bar graph of the quantification of Nrf2 and HO-1 protein levels. Data are expressed as mean ± SEM (n = 3). *P < 0.05. T test was used. **(O)** The representative immunofluorescence images of NLRP3 among the AngII, AngII+C75, and AngII+C75+NAC groups. Scale bar = 100 μm. **(P)** The bar graph of the quantification of fluorescence intensity for NLRP3. Data are expressed as mean ± SEM (n = 4). *P < 0.05, **P < 0.01, ***P < 0.001. One-way ANOVA followed by Tukey’s *post hoc* test was used. **(Q)** The representative WB bands showing the expression of NLRP3, pro-Caspase-1, cle-Caspase-1 and GSDMD-N. **(R–U)** The bar graph of the quantification of NLRP3 **(R)**, pro-Caspase-1 **(S)**, cle-Caspase-1 **(T)** and GSDMD-N **(U)** protein levels. Data are expressed as mean ± SEM (n = 3). Ns means no significance. *P < 0.05, **P < 0.01, ***P < 0.001. One-way ANOVA followed by Tukey’s *post hoc* test was used. **(V, W)** The bar graph of the concentrations of IL-1β **(V)** and IL-18 **(W)** in the cell supernatant. Data are expressed as mean ± SEM (n = 4). **P < 0.01, ***P < 0.001. One-way ANOVA followed by Tukey’s *post hoc* test was used.

C75, an effective Fasn inhibitor, was used to assess its impact on RAOECs. WB analysis revealed that C75 treatment significantly reduced the AngII-induced increase in Fasn protein expression (**Figures 8A, B**). Immunofluorescence staining further confirmed the inhibitory effect of C75 on Fasn expression (**Figures 8C, D**). EdU assays showed a significant enhancement in cell proliferation in the AngII+C75 group (**Supplementary Figure S5A, B**), while flow cytometry revealed a lower apoptotic rate in the AngII+C75 group compared to the AngII group (**Supplementary Figure S5C, D**). WB analysis indicated that VWF and eNOS expression was significantly increased in the AngII+C75 group (**Figures 8E, F**). DHE staining results showed that C75 significantly inhibited ROS production (**Figures 8G, H**). Both immunofluorescence and WB analyses demonstrated upregulated expression of Nrf2 and HO-1 in the AngII+C75 group relative to the AngII group (**Figures 8I–N**). These results suggest that C75 enhanced antioxidant component production and inhibited ROS accumulation. Furthermore, immunofluorescence analysis confirmed that C75 reduced the elevated levels of NLRP3 induced by AngII (**Figures 8O, P**). WB results showed that NAC treatment further promoted the reduction of NLRP3, cle-Caspase-1, GSDMD-N, IL-1β, and IL-18 in the AngII+C75 group, while pro-Caspase-1 expression remained unchanged (**Figures 8Q–W**). These results indicate that C75 reduced ROS production by activating the Nrf2/HO-1 pathway, thereby inhibiting NLRP3 inflammasome-mediated pyroptosis.

### 3.11 C75 improves endothelial function and restores erection function in SHR

**Figure 9:**
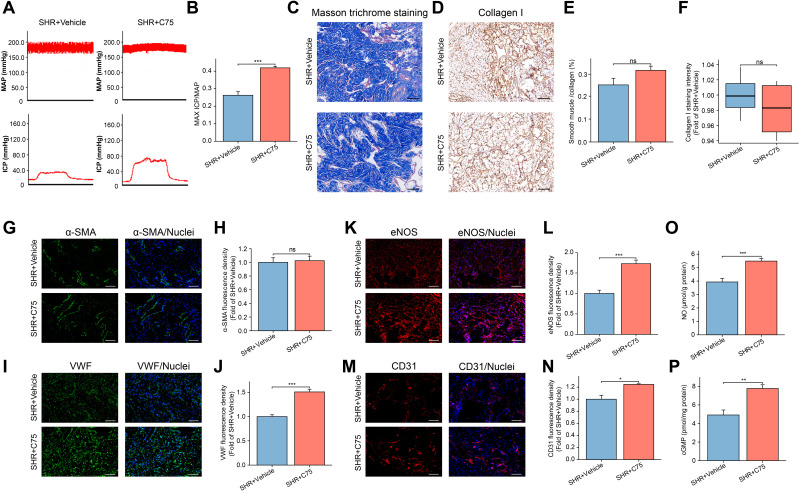
Effects of C75 on erectile function of the SHR group. **(A)** The representative images of ICP and MAP under electrical stimulation of the cavernosal nerve between the SHR+ Vehicle and SHR+C75 groups. **(B)** The bar graph of the maximum ICP/MAP ratio between the two groups. Data are expressed as mean ± SEM (n = 5). ***P < 0.001. T test was used. **(C)** The representative images of Masson’s trichrome staining in the corpus cavernosum tissue. Scale bar = 100 μm. **(D)** The immunohistochemistry staining for Collagen I between the SHR+ Vehicle and SHR+C75 groups. Scale bar = 100 μm. **(E)** The bar graph of the quantification of smooth muscle/collagen content ratio. Data are expressed as mean ± SEM (n = 4). Ns means no significance. T test was used. **(F)** The box graph of the quantification of Collagen I staining intensity. Data are expressed as mean ± SEM (n = 4). Ns means no significance. T test was used. **(G, I, K, M)** The immunofluorescence staining images of α-SMA **(G)**, VWF **(I)**, eNOS **(K)**, and CD31 **(M)** in the corpus cavernosum. Scale bar = 100 μm. **(H, J, L, N)** The bar graph of the quantification of fluorescence intensity for α-SMA **(H)**, VWF **(J)**, eNOS **(L)**, and CD31 **(N)** between the two groups. Data are expressed as mean ± SEM (n = 4). Ns means no significance. *P < 0.05, ***P < 0.001. T test was used. **(O, P)** The bar graph of NO **(O)** and cGMP **(P)** levels between the two groups. Data are expressed as mean ± SEM (n = 6). **P < 0.01, ***P < 0.001. T test was used. SHR, spontaneously hypertensive rats; Norm, normal rats; ICP, intracavernous pressure; MAP, mean arterial blood pressure.

For *in vivo* validation, C75 was intraperitoneally injected into rats. The SHR+C75 group exhibited a significantly higher ICP_max_/MAP ratio compared to the SHR+Vehicle group (**Figures 9A, B**), indicating restored erectile function in SHR. Additionally, C75 treatment increased the expression of VWF, eNOS, and CD31 in penile tissues compared to the SHR+Vehicle group (**Figures 9I–N**). The NO and cGMP levels were also elevated, further supporting the improvement of endothelial function in the penis (**Figures 9O, P**). However, no significant changes were observed in smooth muscle or Collagen I expression (**Figures 9C–H**).

### 3.12 The impact of C75 on the oxidative stress and NLRP3 inflammasome-dependent pyroptosis *in vivo*

**Figure 10:**
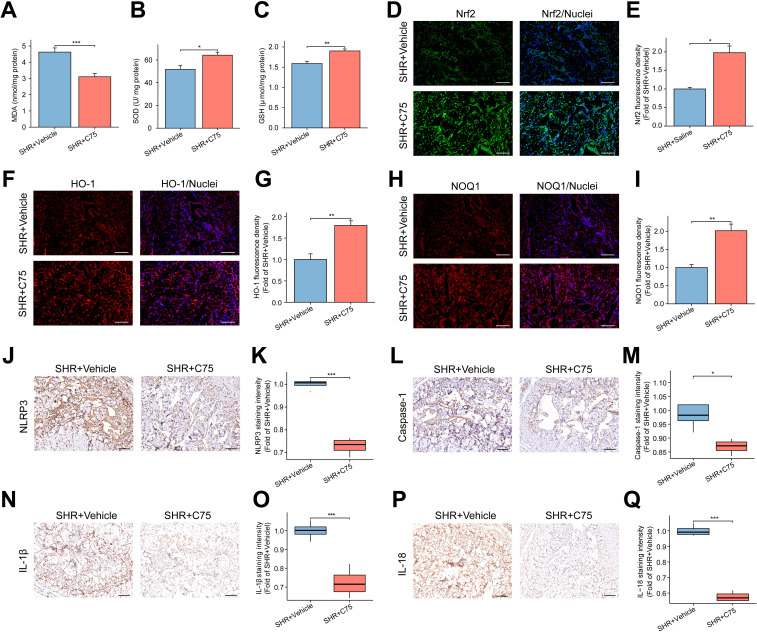
Effects of C75 on oxidative stress and NLRP3 inflammasome-mediated pyroptosis through the Nrf2/HO-1 pathway in the corpus cavernosum. **(A–C)** The bar graph of the content of MDA **(A)**, SOD **(B)** and GSH **(C)** in the corpus cavernosum. Data are expressed as mean ± SEM (n = 5). *P < 0.05, **P < 0.01, ***P < 0.001. T test was used. **(D, F, H)** The representative images of immunofluorescent staining of Nrf2 **(D)**, HO-1 **(F)**, and NOQ1 **(H)** in the corpus cavernosum. Scale bar = 100 μm. **(E, G, I)** The bar graphs of the quantification of fluorescence intensity for Nrf2 **(E)**, HO-1 **(G)**, and NOQ1 **(I)**. Data are expressed as mean ± SEM (n = 4). *P < 0.05, **P < 0.01. T test was used. **(J, L, N, P)**, The representative images of immunohistochemical staining for NLRP3 **(J)**, Caspase-1 **(L)**, IL-1β **(N)**, and IL-18 **(P)** in the corpus cavernosum. Scale bar = 100 μm. **(K, M, O, Q)** The box graphs of the quantification of staining intensity for NLRP3 **(K)**, Caspase-1 **(M)**, IL-1β **(O)**, and IL-18 **(Q)** between the two groups. Data are expressed as mean ± SEM (n = 4). *P < 0.05, ***P < 0.001. T test was used. SHR, spontaneously hypertensive rats; SOD, Superoxide dismutase; MDA, Malondialdehyde; GSH, Glutathione.

In terms of oxidative stress, the SHR+C75 group exhibited significantly lower MDA levels and higher SOD and GSH levels compared to the SHR+Vehicle group (**Figures 10A–C**). Immunofluorescence analysis revealed a significant upregulation of antioxidant stress proteins Nrf2, HO-1, and NOQ1 following C75 treatment (**Figures 10D–I**). Immunohistochemistry confirmed that C75 reduced the expression of NLRP3, Caspase-1, IL-1β, and IL-18 in the penis (**Figures 10J–Q**), suggesting that C75 mitigated pyroptosis through activation of the Nrf2/HO-1 pathway and inhibition of oxidative stress and NLRP3 inflammasome production.

### 3.13 The effect of C75 on apoptosis *in vivo*

**Figure 11:**
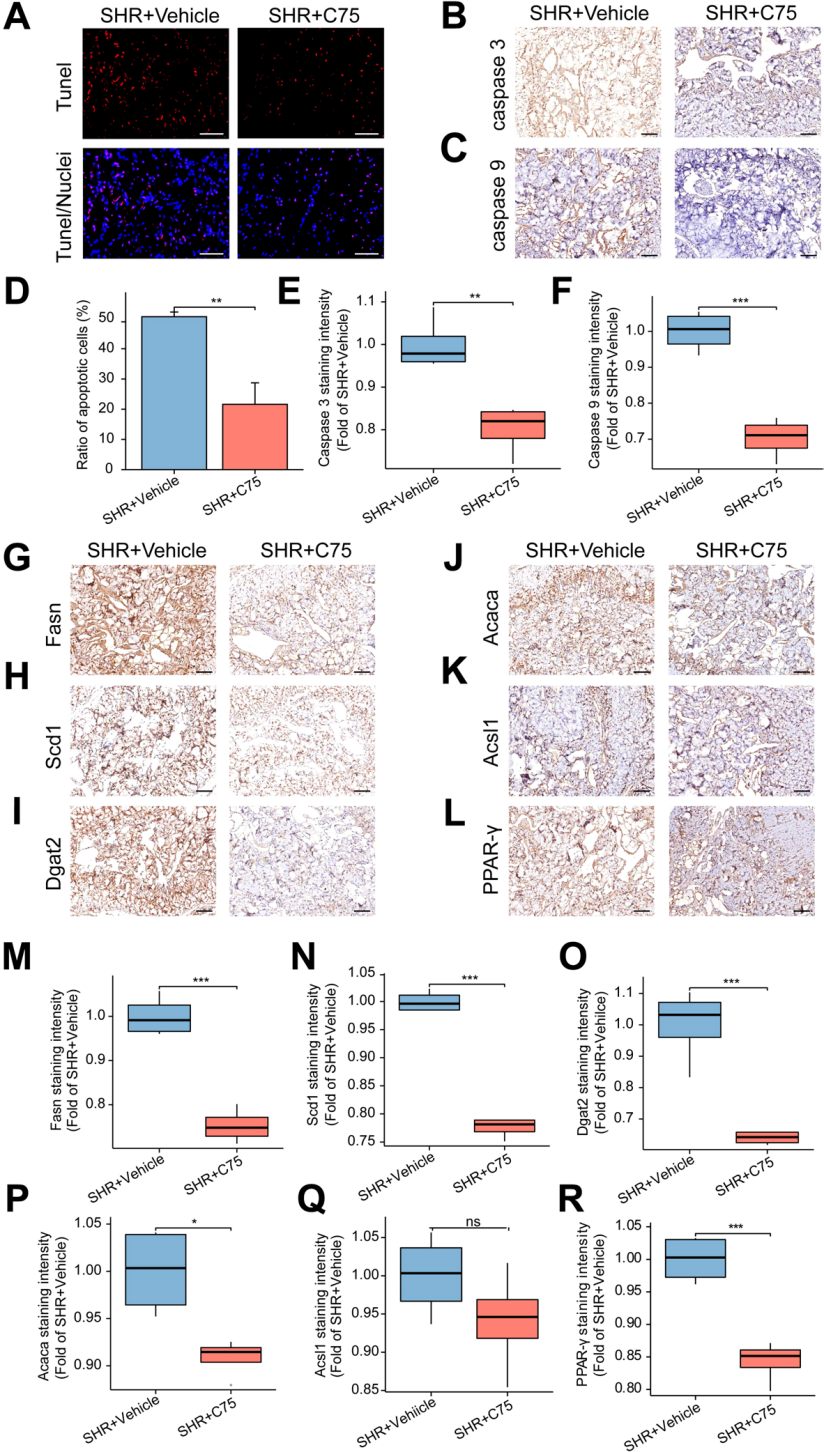
Effects of C75 on apoptosis and the lipid metabolism in the corpus cavernosum of SHR. **(A)** The representative images of TUNEL staining. Scale bar = 100 μm. **(B, C)** The representative images of immunohistochemical staining of Caspase 3 **(B)** and Caspase 9 **(C)**. Scale bar = 100 μm. **(D)** The bar graph of the percentage of TUNEL-positive cells. Data are expressed as mean ± SEM (n = 4). **P < 0.01. T test was used. **(E, F)** The box graphs of the quantitative data of immunohistochemical staining for Caspase 3 **(E)** and Caspase 9 **(F)**. Data are expressed as mean ± SEM (n = 4). **P < 0.01, ***P < 0.001. T test was used. **(G–L)** The representative images of immunohistochemical staining of the lipid metabolism-related genes, including Fasn **(G)**, Scd1 **(H)**, Dgat2 **(I)**, Acaca **(J)** and Acsl1 **(K)**, as well as the core gene PPAR-γ **(L)** in the PPAR signal pathway in the corpus cavernosum. Scale bar = 100 μm. **(M–R)** The box graphs of the quantification of staining intensity for Fasn **(M)**, Scd1 **(N)**, Dgat2 **(O)**, Acaca **(P)**, Acsl1 **(Q)** and PPAR-γ **(R)**. Data are expressed as mean ± SEM (n = 4). Ns means no significance. *P < 0.05, ***P < 0.001. T test was used.

C75 treatment also decreased apoptosis in penile cavernous tissue, as indicated by fewer TUNEL-positive cells in the SHR+C75 group compared to the SHR+Vehicle group (**Figures 11A, D**). Immunohistochemical analysis of Caspase 3 and Caspase 9 further confirmed reduced apoptosis in the SHR+C75 group (**Figures 11B, C, E, F**).

### 3.14 The impact of C75 on the lipid metabolism *in vivo*

Finally, immunohistochemical analysis showed that, except for Acsl1 (**Figures 11K, Q**), the expression of lipid metabolism-related genes Fasn (**Figures 11G, M**), Scd1 (**Figures 11H, N**), Dgat2 (**Figures 11I, O**), Acaca (**Figures 11J, P**), and PPAR-γ (**Figures 11L, R**) were significantly decreased in the SHR+C75 group compared to the SHR+Vehicle group. These results indicated that C75 effectively inhibited lipid metabolism disorders in SHR.

## 4 Discussion

**Figure 12:**
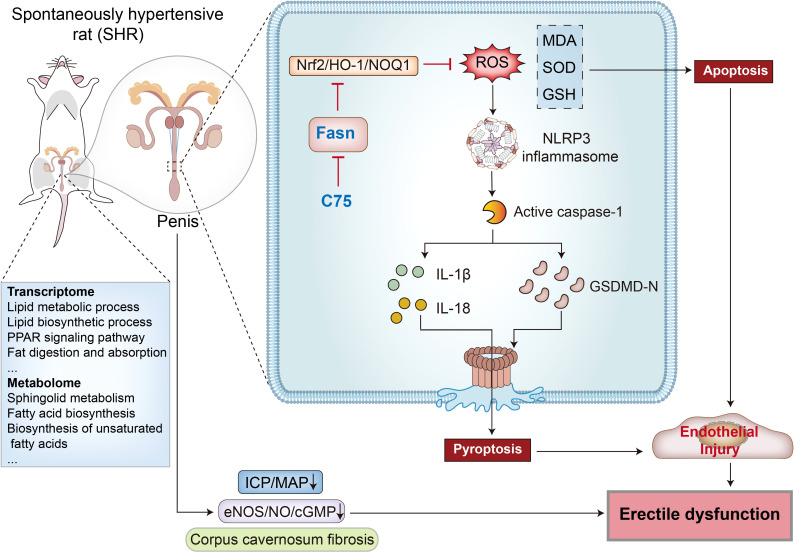
The Diagram illustrates how Fasn inhibition improves erectile dysfunction in spontaneously hypertensive rats. By conducting transcriptomic sequencing and metabolomic sequencing of penile corpus cavernosum tissues, the lipid metabolism pathway and hub gene Fasn were identified. Fasn is involved in the pathological process of hypertension-induced ED through inhibition of the Nrf2/HO-1 pathway, leading to ROS accumulation and NLRP3 inflammasome-mediated pyroptosis. The Fasn inhibitor C75 improves erectile function through reducing oxidative stress and inhibiting NLRP3 inflammasome-dependent pyroptosis via activating the Nrf2/HO-1 signaling pathway.

Hypertension affects approximately 31.1% of adults worldwide and is a leading cause of premature death globally (42). Hypertension increases the risk of ED, which may also serve as an early indicator of hypertension (43). However, the precise mechanisms through which hypertension induces ED remain unclear. This study observed decreased erectile function, elevated fibrosis, and endothelial injury in the penis of SHR. To explore the underlying mechanisms of hypertension-induced ED, transcriptomic and metabolomic analyses were conducted on penile corpus cavernosum tissues. The results highlighted lipid metabolism as the most significant signaling pathway, with Fasn identified as a key hub gene within this pathway. Further investigations revealed that Fasn knockdown or inhibition with C75 activated the Nrf2/HO-1 pathway, mitigating oxidative stress, reducing NLRP3 inflammasome-dependent pyroptosis, and alleviating endothelial cell damage. Ultimately, these interventions restored erectile function (**Figure 12**). These observations may provide novel insights into the treatment of hypertension-induced ED.

Consistent with previous studies linking hypertension to severe ED (44), impaired erectile function was observed in SHR, as indicated by a reduced maximum ICP/MAP ratio. Normal penile erection relies on NO release mediated by NOS, and the imbalance between NO and ROS production in SHR disrupts the NO/cGMP pathway, as confirmed by our results (45). The structural integrity and functional capacity of the corpus cavernosum are essential for penile erection, with vascular endothelial cells serving as the primary component of the sinusoidal vessels in this region, playing a critical role in erectile function. Given that penile erection primarily depends on vascular mechanisms, and endothelial tissue is highly sensitive to both functional and structural changes (43), hypertension-induced ED likely results from alterations in the structure and function of the corpus cavernosum, particularly damage to the vascular endothelial cells. Substantial evidence suggests that endothelial dysfunction is a consequence, rather than a cause, of hypertension (46, 47). A cohort study demonstrated a positive correlation between the severity of hypertension and the extent of endothelial dysfunction (48). In our study, analysis of VWF, CD31, eNOS, and α-SMA expression revealed a loss of endothelial and smooth muscle content in the penises of SHR. While the current studies primarily focus on vascular endothelial dysfunction as central contributors to hypertension-induced ED, neural damage is also a significant factor. As is exhibited that the prevalence of ED after radical prostatectomy for prostate cancer is 85%, with cavernous nerve injury (CNI) as the main cause (49). Animal models of CNI exhibit increased cavernous cell apoptosis due to oxidative stress induced by nerve injury (50). Chronically denervated penile corpus cavernosum exhibits increased fibrosis as well as downregulation of the nNOS/NO pathway (51, 52).

In this study, transcriptomic and metabolomic sequencing analyses were performed on the corpus cavernosum tissue to investigate genetic and metabolic changes associated with hypertension-induced ED. RNAseq results indicated that alterations in lipid metabolism and the PPAR signaling pathway may underlie hypertension-induced ED. Similarly, differential metabolite KEGG enrichment analysis under both anionic and cationic modes also highlighted lipid metabolism pathways, corroborating the RNAseq findings. Moreover, the DAMs also enriched in ABC transporters, arginine biosynthesis, and starch and sucrose metabolism. PPI analysis using the STRING database and Cytoscape software identified Fasn as a central hub gene. The production of Ang II, a key factor in hypertension-induced endothelial dysfunction, is widely used to establish *in vitro* models of hypertension (53). Yin et al. found that Ang II modulates the expression of genes involved in lipid metabolism, including Fasn, thereby promoting atherosclerosis progression (54). Moreover, Ang II-induced changes in lipid levels were observed in the placenta of hypertensive pregnant mice, potentially impairing placental function (55). Excessive apoptosis in cavernous tissue is known to contribute to the development of ED. Our previous study highlighted the involvement of an apoptosis-associated lncRNA-miRNA-mRNA network in ED in CNI rats (56). Carla et al. reported widespread apoptosis in smooth muscle and endothelial cells in diabetic patients with ED (57). In line with these observations, reduced proliferation was observed in the penis of SHR, with *in vitro* Ang II exposure leading to upregulation of Fasn and decreased proliferation in RAOECs. Notably, Fasn knockdown and Fasn inhibition with C75 increased proliferation and reduced apoptosis in RAOECs. Therefore, Fasn likely mediates the inhibitory effect of hypertension on endothelial cell proliferation in the corpus cavernosum.

Hypertension is widely recognized as a condition characterized by oxidative stress and local vascular inflammation (58). Ang II activates the Nox2 enzyme, contributing to hypertension-induced oxidative stress through ROS production (59, 60). In cardiovascular disease progression, areas of vascular damage are typically characterized by elevated local ROS (61). Nrf2 and its downstream target, HO-1, are critical in cellular defense against oxidative stress (62). Under normal conditions, Nrf2 is kept in an inactive state by binding to Keap1 in the cytoplasm (63). Upon activation, phosphorylated Nrf2 translocates to the nucleus, where it binds to AREs and promotes the expression of antioxidant proteins, including HO-1 and NQO1 (64, 65). Consistent with our hypothesis, treatment with siFasn and C75 upregulated Nrf2 and HO-1 expression in Ang II-induced RAOECs, reversing the elevated ROS levels.

ROS is widely acknowledged as an upstream mediator that promotes the activation of the NLRP3 inflammasome, triggering subsequent inflammatory cascades (66). Activation of the NLRP3 inflammasome leads to the recruitment and cleavage of pro-Caspase-1, followed by the activation of IL-1β and IL-18, ultimately inducing pyroptosis (67). In this study, siFasn and C75 significantly reduced the protein expression of NLRP3 and GSDMD-N in Ang II-induced RAOECs. To investigate whether the suppressive effect of Fasn inhibition on NLRP3 inflammasome-dependent pyroptosis is mediated through the reduction of ROS production, NAC, an antioxidant that eliminates ROS by promoting GSH synthesis, was used (68). Following ROS inhibition, the expression of GSDMD-N, NLRP3, IL-1β, and IL-18 was notably reduced. These results suggest that Fasn inhibition reduces ROS levels *via* the Nrf2/HO-1 pathway, thereby preventing NLRP3 inflammasome activation and mitigating the inflammatory response and cell pyroptosis induced by Ang II. Additionally, *in vivo* rescue experiments showed that treatment with the Fasn inhibitor C75 in SHR increased the expression of endothelial markers, including VWF, CD31, and eNOS, in the penis. These findings further suggest that C75 improved endothelial function, leading to the restoration of erectile function. Notably, no significant changes were observed in collagen I and α-SMA expression, reinforcing the idea that Fasn inhibitors primarily promote repair of the vascular endothelium in the penile corpus cavernosum, rather than affecting smooth muscle or fibrotic processes. C75 has been shown to enhance neutrophil chemotaxis by inhibiting Fasn, and it also improves survival of mice with sepsis in septic shock models (69). Singh et al. revealed that intraperitoneal injection of C75 in monocrotaline treated rats reduces right ventricular hypertrophy and improves cardiac function associated with pulmonary hypertension by enhancing metabolic function (70). Additionally, C75 induces apoptosis across various cancer cell lines, suggesting its potential as a therapeutic target for cancer (71). However, C75 can inhibit the mitochondrial fatty acid synthesis pathway, thereby impairing mitochondrial function (72). Some studies indicate that C75 may influence metabolic pathways beyond fatty acid synthesis, such as enhanced β-oxidation, and these effects have been linked to weight loss and anorexia nervosa (73, 74). While C75 presents certain adverse effects, these appear to be acceptable given its remarkable therapeutic efficacy.

This study represents one of the first to explore the role of lipid metabolism in hypertension-induced ED in SHR. Our results underscore the critical involvement of Fasn in oxidative stress and pyroptosis, highlighting the potential therapeutic value of C75 in modulating these pathways. However, certain limitations should be noted. While hypertension induces ED through the classical pyroptosis pathway, the contribution of non-classical pathways remains unexplored. Moreover, although the focus of our study was on vascular endothelial damage, the potential role of neural damage in ED was not extensively examined. Besides, for future studies, it is essential to use larger sample sizes to validate our findings.

## 5 Conclusion

In summary, a comprehensive analysis of transcriptomics and metabolomics revealed that lipid metabolism plays a pivotal role in hypertension-induced ED. Fasn inhibition was shown to reduce endothelial damage and functional changes by suppressing oxidative stress and NLRP3 inflammasome-dependent pyroptosis through activation of the Nrf2/HO-1 pathway. Furthermore, in SHR, the pharmacological inhibition of Fasn by C75 improved endothelial function and ameliorated ED. Therefore, C75 represents a promising therapeutic target for hypertension-induced ED.

## Data Availability

The datasets presented in this study can be found in online repositories. The names of the repository/repositories and accession number(s) can be found below: GSE285267 (GEO).

## Supplementary material

Supplementary Figure 1 The bar graph of the levels of MDA **(A)**, SOD **(B)** and GSH **(C)** in the corpus cavernosum between the norm and SHR groups. Data are expressed as mean ± SEM (n = 5). ***P < 0.001. T test was used. SHR: spontaneously hypertensive rats, Norm: normal rats,

Supplementary Figure 2 The results of response permutation tests in the positive ionization mode **(A)** and negative ionization mode **(B)**,

Supplementary Figure 3 The GSEA results revealing gene enrichment in PPAR signaling pathway **(A)** and fatty acid degradation **(B)**,

Supplementary Figure 4
**(A)** Cell viability was measured by CCK8 reagents under different concentrations of Ang II in RAOECs. Data are expressed as mean ± SEM (n = 5). Ns means no significance. **P < 0.01, ***P < 0.001. One-way ANOVA followed by Tukey’s *post hoc* test was used. **(B)** The colony formation assay in RAOECs. **(C)** The bar graph of the numbers of colonies. Data are expressed as mean ± SEM (n = 5). **P < 0.01, ***P < 0.001. One-way ANOVA followed by Tukey’s *post hoc* test was used.

Supplementary Figure 5
**(A)** The representative images of EdU assays between the AngII and AngII+C75 groups. Scale bar = 200 μm. **(B)** The bar graph of the proliferation rate between the two groups. Data are expressed as mean ± SEM (n = 4). **P < 0.01. T test was used. **(C)** The representative images of flow cytometry analysis of apoptosis between the AngII and AngII+C75 groups. **(D)** The flow cytometry analysis showing the apoptosis rate in RAOECs. Data are expressed as mean ± SEM (n = 3). ***P < 0.001. T test was used.
