# Supplementary figures and images for: Fatty acid synthase inhibition improves hypertension-induced erectile dysfunction by suppressing oxidative stress and NLRP3 inflammasome-dependent pyroptosis through activating the Nrf2/HO-1 pathway
Source: Front Immunol. 2025 Jan 14;15:1532021. doi: 10.3389/fimmu.2024.1532021 (PMC11772187; doi:10.3389/fimmu.2024.1532021)

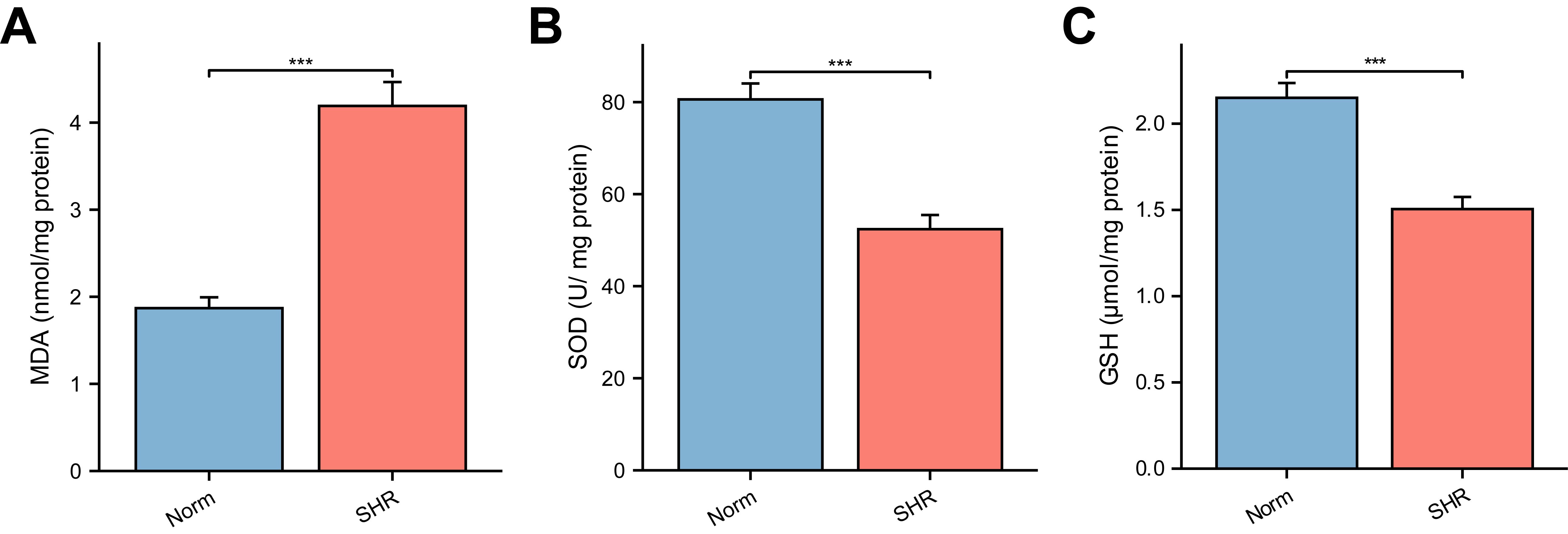

Supplement: Supplementary Figure 1 — The bar graph of the levels of MDA (A), SOD (B) and GSH (C) in the corpus cavernosum between the norm and SHR groups. Data are expressed as mean ± SEM (n = 5). ***P < 0.001. T test was used. SHR: spontaneously hypertensive rats, Norm: normal rats, [file Image1.jpeg]

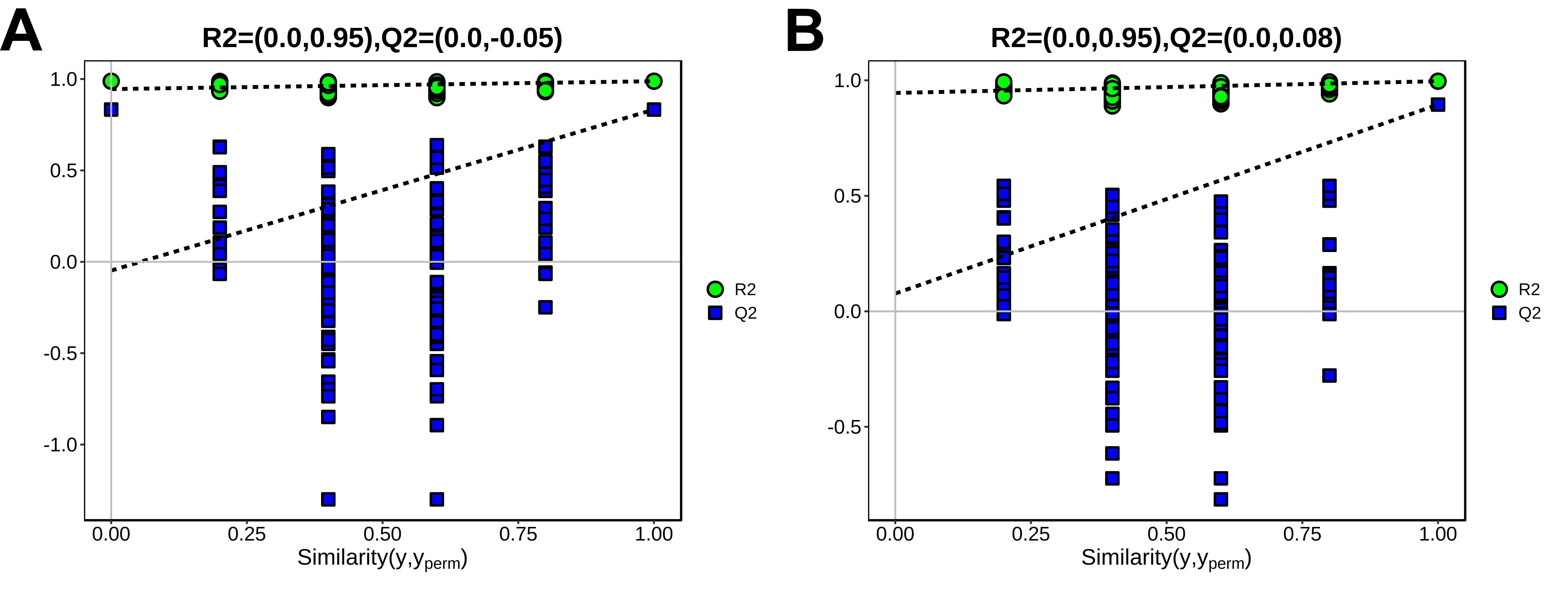

Supplement: Supplementary Figure 2 — The results of response permutation tests in the positive ionization mode (A) and negative ionization mode (B), [file Image2.jpeg]

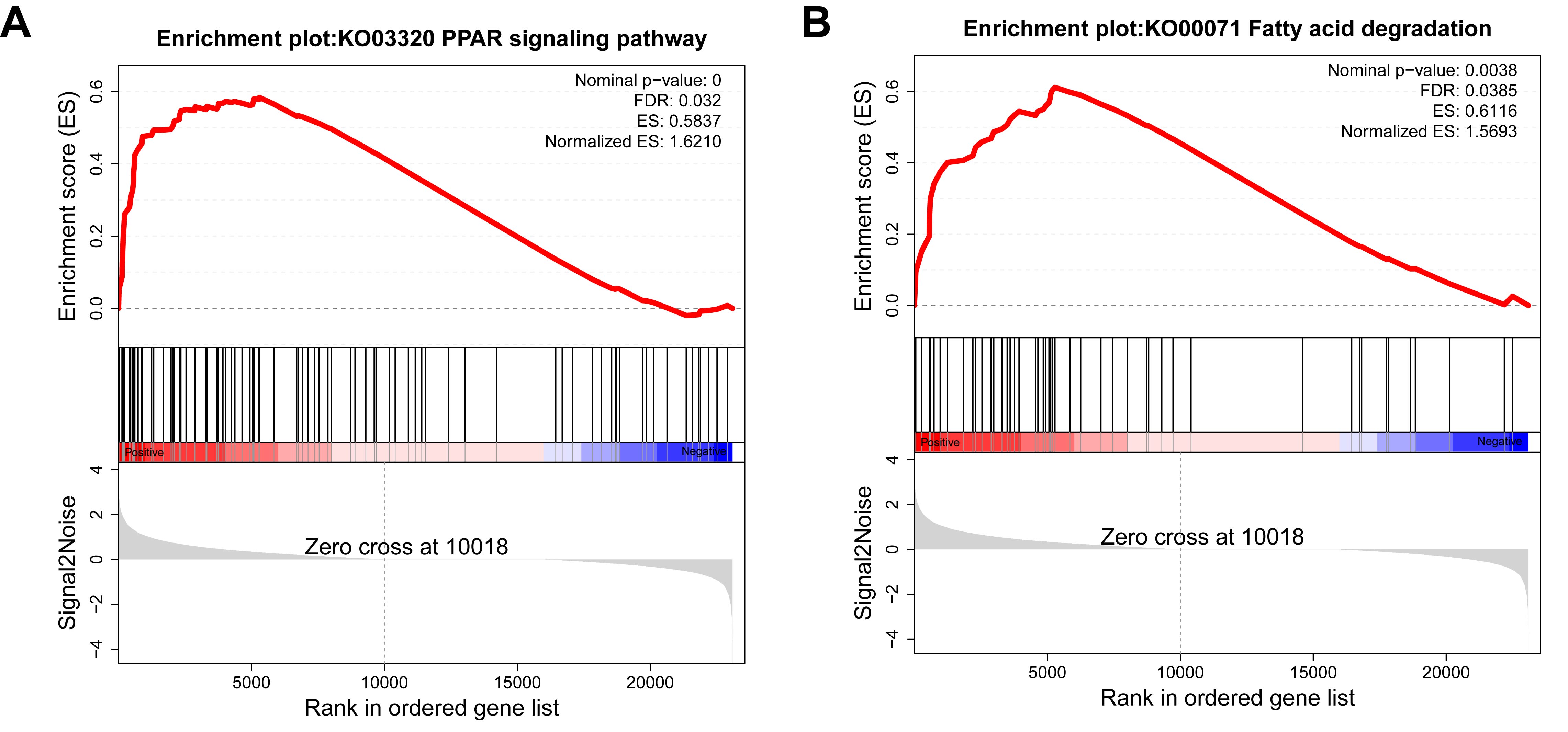

Supplement: Supplementary Figure 3 — The GSEA results revealing gene enrichment in PPAR signaling pathway (A) and fatty acid degradation (B), [file Image3.jpeg]

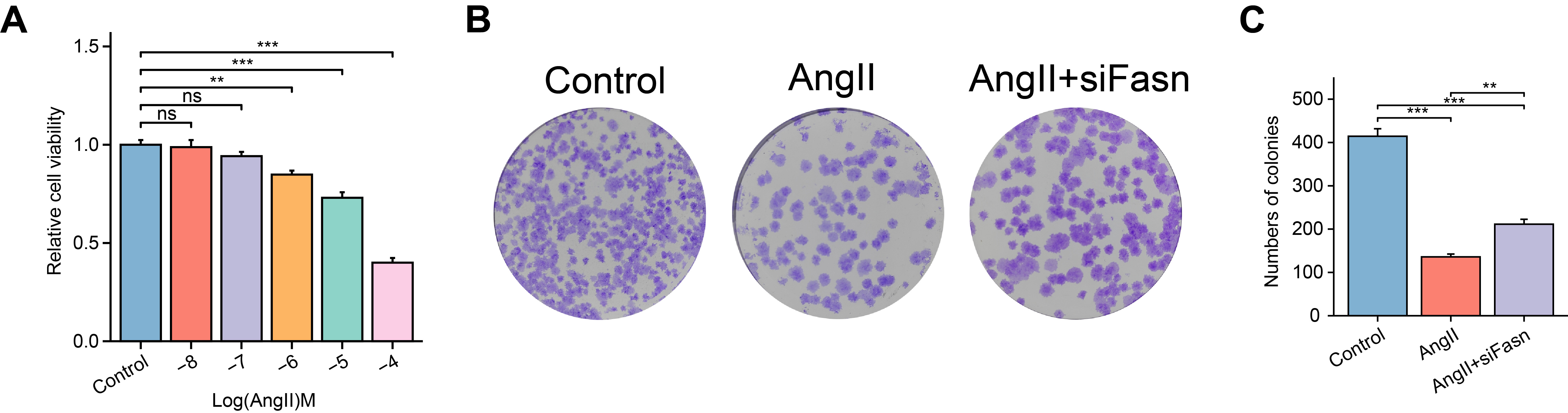

Supplement: Supplementary Figure 4 — (A) Cell viability was measured by CCK8 reagents under different concentrations of Ang II in RAOECs. Data are expressed as mean ± SEM (n = 5). Ns means no significance. **P < 0.01, ***P < 0.001. One-way ANOVA followed by Tukey’s post hoc test was used. (B) The colony formation assay in RAOECs. (C) The bar graph of the numbers of colonies. Data are expressed as mean ± SEM (n = 5). **P < 0.01, ***P < 0.001. One-way ANOVA followed by Tukey’s post hoc test was used. [file Image4.jpeg]

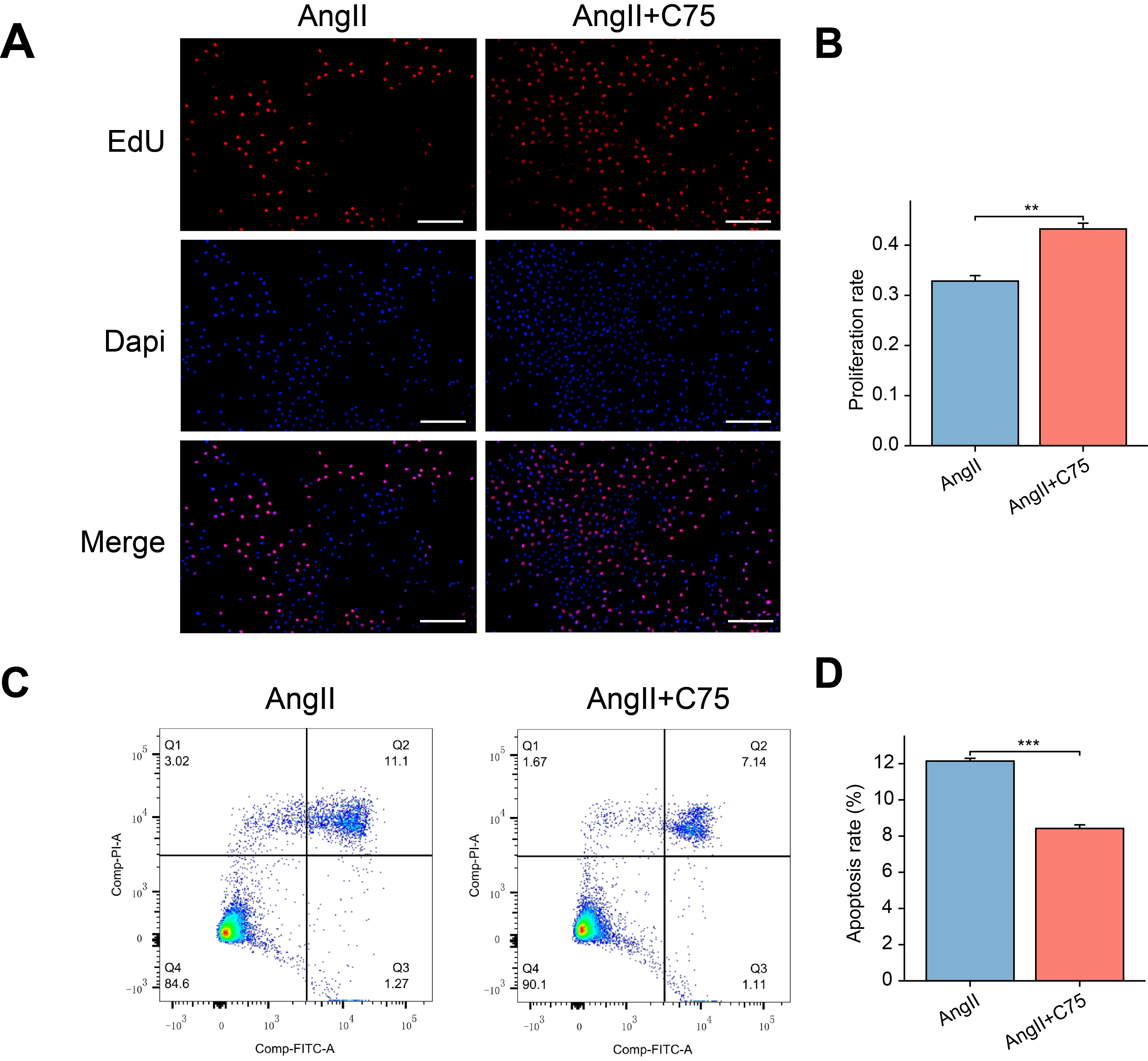

Supplement: Supplementary Figure 5 — (A) The representative images of EdU assays between the AngII and AngII+C75 groups. Scale bar = 200 μm. (B) The bar graph of the proliferation rate between the two groups. Data are expressed as mean ± SEM (n = 4). **P < 0.01. T test was used. (C) The representative images of flow cytometry analysis of apoptosis between the AngII and AngII+C75 groups. (D) The flow cytometry analysis showing the apoptosis rate in RAOECs. Data are expressed as mean ± SEM (n = 3). ***P < 0.001. T test was used. [file Image5.jpeg]
