# Supplementary material for: Fatty acid synthase inhibition improves hypertension-induced erectile dysfunction by suppressing oxidative stress and NLRP3 inflammasome-dependent pyroptosis through activating the Nrf2/HO-1 pathway
Source: Front Immunol. 2025 Jan 14;15:1532021. doi: 10.3389/fimmu.2024.1532021 (PMC11772187; doi:10.3389/fimmu.2024.1532021)
Supplement: Supplementary file 6 [file Table1.docx]

Table S1. PCR primers used in the research.

| **Primers** |  | **Sequence (5’-3’)** |
| --- | --- | --- |
| **Fasn**  **GAPDH** | Forward  Reverse  Forward  Reverse | GGAGGTGGTGATAGCCGGTAT  TGGGTAATCCATAGAGCCCAG  GGCACAGTCAAGGCTGAGAATG  ATGGTGGTGAAGACGCCAGTA |
