# Supplementary material for: Fatty acid synthase inhibition improves hypertension-induced erectile dysfunction by suppressing oxidative stress and NLRP3 inflammasome-dependent pyroptosis through activating the Nrf2/HO-1 pathway
Source: Front Immunol. 2025 Jan 14;15:1532021. doi: 10.3389/fimmu.2024.1532021 (PMC11772187; doi:10.3389/fimmu.2024.1532021)
Supplement: Supplementary file 7 [file Table2.docx]

**Table S2. siRNA sequences.**

|  | Sequences (5’-3’) |
| --- | --- |
| si-Fasn-1  si-Fasn-2  si-Fasn-3 | CGGCUCUCUUUCUUCUUCGACUUCA  UGAAGUCGAAGAAGAAAGAGAGCCG  GACUUGGCCUUUGUGAGCAUGCUCA  UGAGCAUGCUCACAAAGGCCAAGUC  CAGCUAAAGCAAGAGGGCGUGUUUG  CAAACACGCCCUCUUGCUUUAGCUG |
