# Supplementary material for: Fatty acid synthase inhibition improves hypertension-induced erectile dysfunction by suppressing oxidative stress and NLRP3 inflammasome-dependent pyroptosis through activating the Nrf2/HO-1 pathway
Source: Front Immunol. 2025 Jan 14;15:1532021. doi: 10.3389/fimmu.2024.1532021 (PMC11772187; doi:10.3389/fimmu.2024.1532021)
Supplement: Supplementary file 8 [file Table3.docx]

**Table S3. Differential expressed POS metabolites between the normal and the spontaneously hypertensive rats (SHR) groups.**

| **Compound_ID** | **Name** | **P value** | **Log_2_FC** | **Vip** |
| --- | --- | --- | --- | --- |
| Com_1137_pos  Com_1772_pos  Com_3997_pos  Com_6424_pos  Com_4546_pos  Com_4102_pos  Com_5728_pos  Com_3847_pos  Com_1307_pos  Com_6252_pos  Com_3273_pos  Com_1649_pos  Com_629_pos  Com_3700_pos  Com_6009_pos  Com_6285_pos  Com_7387_pos  Com_2114_pos  Com_2039_pos  Com_8129_pos  Com_5654_pos  Com_1916_pos  Com_5176_pos  Com_357_pos  Com_2662_pos  Com_2282_pos  Com_5928_pos  Com_7631_pos  Com_6467_pos  Com_5061_pos  Com_189_pos  Com_6541_pos  Com_1390_pos  Com_6851_pos  Com_3661_pos  Com_3571_pos  Com_1963_pos  Com_1209_pos  Com_3726_pos  Com_3084_pos  Com_129_pos  Com_370_pos  Com_1818_pos  Com_28_pos  Com_2087_pos  Com_4531_pos  Com_1254_pos  Com_156_pos  Com_4463_pos  Com_191_pos  Com_1548_pos  Com_50_pos  Com_2271_pos  Com_3756_pos  Com_389_pos  Com_4182_pos  Com_1596_pos  Com_292_pos  Com_408_pos  Com_1529_pos  Com_7043_pos  Com_312_pos  Com_6750_pos  Com_2076_pos  Com_108_pos  Com_1471_pos  Com_2205_pos  Com_1509_pos  Com_558_pos  Com_3397_pos  Com_3275_pos  Com_2069_pos  Com_1982_pos  Com_377_pos  Com_293_pos  Com_7073_pos  Com_2338_pos  Com_450_pos  Com_268_pos  Com_6615_pos  Com_4405_pos  Com_76_pos  Com_2746_pos  Com_5457_pos  Com_394_pos  Com_4350_pos  Com_5290_pos  Com_49_pos  Com_7253_pos  Com_4703_pos  Com_513_pos  Com_1313_pos  Com_517_pos  Com_178_pos  Com_57_pos  Com_2162_pos  Com_440_pos  Com_3291_pos  Com_4871_pos  Com_7514_pos  Com_4344_pos  Com_2684_pos  Com_140_pos  Com_6253_pos  Com_1375_pos  Com_3866_pos  Com_325_pos  Com_2973_pos  Com_3594_pos  Com_1191_pos  Com_6970_pos  Com_4174_pos  Com_1179_pos  Com_7790_pos  Com_2034_pos  Com_300_pos  Com_2268_pos  Com_4695_pos  Com_2387_pos  Com_1835_pos  Com_1559_pos  Com_2096_pos  Com_4679_pos  Com_694_pos  Com_2201_pos  Com_2552_pos  Com_287_pos  Com_8369_pos  Com_9121_pos  Com_3327_pos  Com_726_pos  Com_899_pos  Com_5774_pos  Com_8119_pos  Com_225_pos  Com_5066_pos  Com_228_pos  Com_6828_pos  Com_3935_pos  Com_2734_pos  Com_2432_pos  Com_1221_pos  Com_3928_pos  Com_2573_pos  Com_1208_pos  Com_5350_pos  Com_990_pos  Com_4968_pos  Com_6761_pos  Com_4228_pos  Com_1957_pos  Com_3959_pos  Com_4186_pos  Com_4045_pos  Com_3075_pos  Com_486_pos  Com_3223_pos  Com_1045_pos  Com_7815_pos  Com_260_pos  Com_1377_pos  Com_1369_pos  Com_3426_pos  Com_278_pos  Com_571_pos  Com_4090_pos  Com_9502_pos | Desmethylcitalopram  Desoxycortone  C-6 NBD ceramide  3'-O-Methylinosine  1-[2-(2,5-dimethyl-1H-pyrrol-1-yl)-4-nitrophenyl]-1H-imidazole  SMH  PC (11:0/13:1)  N1-(1H-indol-4-yl)cyclohexane-1-carboxamide  Desloratadine  2-{[(4,5-dimethoxy-2-nitrophenethyl)imino]methyl}phenol  16-Heptadecyne-1,2,4-triol  Gibberellic acid  1,7-bis(3,4-dihydroxyphenyl)heptan-3-one  8,8-dimethyl-2-phenyl-4H,8H-pyrano[2,3-h]chromen-4-one  Neosaxitoxin  Phytosphingosine  3-(3,4-dimethoxyphenyl)-1-(2-hydroxy-4,6-dimethoxyphenyl)propan-1-one  Quinoline  Argininosuccinic acid  Gly-Tyr-Ala  CGS 12066B  Isoliquiritigenin  Medroxyprogesterone  Glycerophospho-N-palmitoyl ethanolamine  11β-Hydroxyandrosterone  15-Deoxy-Δ12,14-prostaglandin A1  N-[1-(4-methoxy-2-oxo-2H-pyran-6-yl)-2-methylbutyl]acetamide  1-(7-methoxy-2-oxo-2H-chromen-8-yl)-3-methyl-2-oxobutyl acetate  N'-[6-(tert-butyl)thieno[3,2-d]pyrimidin-4-yl]-4-methylbenzohydrazide  Sinefungin  L-Aspartic acid  2-{[4-(tert-butyl)benzyl]thio}-4-methylquinoline-3-carbonitrile  (±)11(12)-DiHET  AKK  PC (18:5e/2:0)  Isotretinoin  5-OxoETE  Eicosapentaenoic acid  4-Deacetylneosolaniol  6ß-Hydroxytestosterone  all-cis-4,7,10,13,16-Docosapentaenoic acid  3-Ureidopropionic acid  Phenylacetylglycine  D-(-)-Glutamine  11-Deoxy prostaglandin F1β  15-Acetyldeoxynivalenol  10-Nitrolinoleate  8Z,11Z,14Z-Eicosatrienoic acid  Coproporphyrin III tetramethyl ester  PC (18:4e/2:0)  PC (18:3e/2:0)  L-Glutamic acid  Diethyl maleate  Testosterone  Xanthurenic acid  PC (16:0e/3:0)  SB 205607  Creatine phosphate  L-serine  Bafilomycin B1  3,4-dihydro-2H-benzo[4,5]imidazo[2,1-b][1,3]thiazin-3-ol  DL-Stachydrine  ACar 20:2  2-(2-thienyl)-4H-chromen-4-one  Caprolactam  Prolylglycine  2,4,5-Trimethoxybenzaldehyde  RKK  O-Phosphocolamine  bicyclo[2.2.2]oct-2-en-1-yl 4-methylbenzene-1-sulfonate  JWH 018 N-pentanoic acid metabolite  ACar 20:4  ACar 18:2  trans-4-Hydroxy-L-proline  2-(3,5-dimethyl-1H-pyrazol-4-yl)-3-nitropyridine  ACar 15:0  3,5-Dimethoxybenzoic acid  Glucose 1-phosphate  Pyridoxamine  PC (14:1e/3:0)  Etiocholanolone  L-Pyroglutamic acid  (±)13-HpODE  Dihydromethysticin  4-(trifluoromethyl)nicotinic acid  O3-(2-thienylcarbonyl)-5-methyl-2-phenyl-3-furancarbohydroximamide  Pregnenolone  Proline  ACar 20:3  Prostaglandin B1  16(R)-HETE  PC (18:4e/4:0)  LPC 18:5  DLK  Docosapentaenoic acid  1-(3-ethyl-2,4-dihydroxy-6-methoxyphenyl)butan-1-one  1,4-dihydroxyheptadec-16-en-2-yl acetate  TNK  N1-(1,3-thiazolan-2-yliden)-3,5-dichloroaniline  Pheniramine N-oxide  5-Methyltetrahydrofolic acid  7,8-Dihydroneopterin  L-Histidine  N-[2,5-bis(2,2,2-trifluoroethoxy)benzoyl]-N'-(4-methoxyphenyl)urea  Isoquinoline  dUMP  3-methyl-5-oxo-5-(4-toluidino)pentanoic acid  Thiamine  Guanosine monophosphate  N-acetyl-D-glucosamine  PC (4:0/16:2)  Senecionine  2,3-dihydroxypropyl 12-methyltridecanoate  Lapachol  2-Myristoylglycerol  (2E,4E)-N-[2-(4-hydroxyphenyl)ethyl]dodeca-2,4-dienamide  Propionyl-L-carnitine  16α-Hydroxydehydroepiandrosterone  2-Methoxybenzaldehyde  Retrorsine  2-Arachidonoyl glycerol  5-Methylcytosine  1,2-dihydroxyheptadec-16-yn-4-yl acetate  2'-Deoxycytidine  Ethyl paraben  3-(4-hydroxy-3-methoxyphenyl)propanoic acid  5(S),15(S)-DiHETE  5-fluoro AB-PINACA N-(4-hydroxypentyl) metabolite  2-hydroxy-3,6-diphenylcyclohexyl acetate  Cerivastatin  o-Cresol  Palmitoylcarnitine  N-(cyclopropylmethyl)-N'-phenylurea  ACar 22:6  D-(+)-Maltose  4-Amino-6-chloro-1,3-benzenedisulfonamide  5-nitro-2-furaldehyde 2,2-dimethylhydrazone  Pyridoxal  5-(hydroxymethyl)-4-methoxy-2,5-dihydrofuran-2-one  5-Fluoro-2-[(3S)-1-(2-methylbenzyl)-3-pyrrolidinyl]-1H-benzimidazole  3,4-Dimethylbenzoic acid  Thiophene-2-carbaldehyde O-2-(2,6-dichlorobenzyl)oxime  allantoate  Flavin mononucleotide (FMN)  2-[5-(2-hydroxypropyl)oxolan-2-yl]propanoic acid  Heptadecanoic Acid  N'1-(6-methylpyridazin-3-yl)-4-chlorobenzene-1-carbohydrazide  5,6-dimethyl-4-oxo-4H-pyran-2-carboxylic acid  2-(2,6-dimethoxyphenyl)-5,6-dimethoxy-4H-chromen-4-one  Cortisone  Cytidine 5'-monophosphate (hydrate)  Oleoyl ethanolamide  Epitestosterone  DL-5-Methoxytryptophan  Biotin  Maltotetraose  ACar 18:0  α-Methyl-DL-histidine  PC (16:0/14:1)  PC (16:2e/2:0)  T-2 Triol  N-Acetylornithine  N-Carbamyl-L-glutamicacid  Dehydrocholic acid  5α-Tetrahydrocortisol  N'-{6-[(5-chloro-3-pyridyl)oxy]-3-pyridyl}-N,N-dimethyliminoformamide  Cannabigerolic acid | 1.25E-06  1.57E-06  1.69E-06  5.77E-06  1.38E-05  1.80E-05  2.78E-05  4.04E-05  4.49E-05  3.23E-05  3.58E-05  4.92E-05  5.63E-05  5.32E-05  7.58E-05  6.84E-05  0.000268097  0.000147735  0.000124501  0.00011903  0.00011044  9.08E-05  8.95E-05  0.000281904  0.000221147  0.000149475  0.000236766  0.000207168  0.00024809  0.000237398  0.000233448  0.000326837  0.000492413  0.001181179  0.000718425  0.000849292  0.000503773  0.000657157  0.00048049  0.000487356  0.000985039  0.000695601  0.001328378  0.001025475  0.001801448  0.001925886  0.001278341  0.001386007  0.002158572  0.002232731  0.002888697  0.001305515  0.002336731  0.003061744  0.001876003  0.002580061  0.001692136  0.003346962  0.001798437  0.007554637  0.008329422  0.003437459  0.002925203  0.004982983  0.002704687  0.00584447  0.005379345  0.009278777  0.002252916  0.006976695  0.003957261  0.005677691  0.004591776  0.003045608  0.004216512  0.005912785  0.006828749  0.005622228  0.009236723  0.006730734  0.010124603  0.006691873  0.00507667  0.004949253  0.009163192  0.008228869  0.009681505  0.006439089  0.011432097  0.005630611  0.008815616  0.010359855  0.009371844  0.013610922  0.009356837  0.014308047  0.01488363  0.01631349  0.014626064  0.00744033  0.01612569  0.006425006  0.011082379  0.011258189  0.014608475  0.014443208  0.022192036  0.010707909  0.010438852  0.012314297  0.015575908  0.012920485  0.019066321  0.013717715  0.019704753  0.032059501  0.022934621  0.015021714  0.019355784  0.012687652  0.017093066  0.026372089  0.015781758  0.028000484  0.020538866  0.018137736  0.026847127  0.009310652  0.03036006  0.021682475  0.023442266  0.016208632  0.021084595  0.02828078  0.010787925  0.034163899  0.012995847  0.03914852  0.036806777  0.036495435  0.043389475  0.02358127  0.031820542  0.045067573  0.027437027  0.041535076  0.026329016  0.033988356  0.03713514  0.019690174  0.023531508  0.03165249  0.034043804  0.046471149  0.042787717  0.02748458  0.02525013  0.047915743  0.036079477  0.04006802  0.026946764  0.042290274  0.0445802  0.0293638  0.032599537  0.040489257  0.039157009 | \| 4.33650016 \| \| --- \| \| 4.459969623 \| \| 3.726251768 \| \| 2.892210932 \| \| 3.822126857 \| \| 2.097859142 \| \| 1.556461513 \| \| 3.381614574 \| \| 3.569246972 \| \| 1.351263297 \| \| 1.360319131 \| \| 2.06294362 \| \| 2.024133999 \| \| 3.218928067 \| \| 2.902295131 \| \| 2.884617203 \| \| 1.515467062 \| \| -1.344248051 \| \| 1.962556865 \| \| 2.555893997 \| \| 2.825102871 \| \| 4.832958329 \| \| 3.816178267 \| \| 1.013025161 \| \| 3.065868776 \| \| 1.2430442 \| \| 1.184020944 \| \| 2.787416782 \| \| 1.748492315 \| \| 2.562788829 \| \| 0.677609915 \| \| 0.90132022 \| \| 1.826438114 \| \| 0.354860121 \| \| 2.11538649 \| \| 2.286212212 \| \| 1.451022585 \| \| 2.006593076 \| \| 2.247521446 \| \| 1.652619586 \| \| 1.302835092 \| \| 0.645677116 \| \| -1.8847737 \| \| 1.076706275 \| \| 1.386599511 \| \| 2.04481623 \| \| -0.81147655 \| \| 0.873829481 \| \| -1.171731189 \| \| 1.17887796 \| \| 1.498817082 \| \| 0.520639555 \| \| -0.609409865 \| \| 1.102094964 \| \| 1.468052383 \| \| -0.851015499 \| \| 0.892042794 \| \| -1.279551781 \| \| 0.367196949 \| \| -1.104701847 \| \| -1.859200868 \| \| 0.721131199 \| \| -1.534872038 \| \| -0.727950543 \| \| 2.419418383 \| \| 0.855922564 \| \| -0.574634481 \| \| -1.051770848 \| \| 0.411156416 \| \| 3.270112556 \| \| 0.486286267 \| \| -0.887183056 \| \| -1.565242828 \| \| 0.845621565 \| \| -0.668684526 \| \| -0.955842696 \| \| -0.53781562 \| \| 0.61223218 \| \| -0.434090452 \| \| 0.618912144 \| \| 0.722172051 \| \| 0.890201302 \| \| 0.853215952 \| \| 1.223759366 \| \| -0.745686173 \| \| -2.04409707 \| \| 1.876671377 \| \| 0.704418607 \| \| -1.226477627 \| \| 0.704173164 \| \| 0.846200059 \| \| 0.866812173 \| \| 3.40804618 \| \| 0.578870245 \| \| 0.812043332 \| \| -0.703152038 \| \| 3.008971869 \| \| -0.503729946 \| \| 1.23252971 \| \| 1.90218893 \| \| 0.818835287 \| \| 0.903113747 \| \| 1.134237404 \| \| -0.647212573 \| \| -0.479956124 \| \| 1.567703169 \| \| -0.873944865 \| \| 0.711452689 \| \| 1.114040638 \| \| 0.546590914 \| \| 1.03068392 \| \| 0.591078148 \| \| 1.842779696 \| \| -1.01766838 \| \| 1.732349191 \| \| -0.631640847 \| \| -0.630852031 \| \| 0.830169388 \| \| -0.536861268 \| \| -0.819002655 \| \| 0.874360264 \| \| -0.588787692 \| \| 3.048582473 \| \| -0.926272549 \| \| -0.535154375 \| \| -0.447746205 \| \| 0.694351066 \| \| 0.649157809 \| \| 1.30814867 \| \| 2.670224311 \| \| -0.209479055 \| \| -0.8982111 \| \| 0.602749494 \| \| -0.83766817 \| \| 0.57261088 \| \| 0.908945501 \| \| 0.436703198 \| \| -0.408810427 \| \| -0.680432112 \| \| -0.507317445 \| \| -0.465198394 \| \| -0.48439213 \| \| 0.960942207 \| \| -0.86356006 \| \| -0.480259963 \| \| 0.780331082 \| \| 1.517580765 \| \| -0.581500928 \| \| -0.561839946 \| \| 0.773814423 \| \| 0.549544948 \| \| 1.36263406 \| \| 0.587705112 \| \| -0.491375876 \| \| -0.38129803 \| \| 0.857799917 \| \| -1.092337037 \| \| -0.799312674 \| \| 0.951542711 \| \| 0.763097387 \| \| 0.746421003 \| \| 0.453296769 \| \| 0.287731207 \| \| -0.477210821 \| \| -0.595257478 \| \| -0.399309398 \| \| 0.501487608 \| | \| 1.892972714 \| \| --- \| \| 1.888150773 \| \| 1.880406032 \| \| 1.85810804 \| \| 1.84682196 \| \| 1.84323178 \| \| 1.839020539 \| \| 1.835471405 \| \| 1.832563233 \| \| 1.831432511 \| \| 1.824177375 \| \| 1.823926798 \| \| 1.820132002 \| \| 1.816642469 \| \| 1.811277674 \| \| 1.808674228 \| \| 1.805492458 \| \| 1.804923005 \| \| 1.800053225 \| \| 1.795983961 \| \| 1.791874539 \| \| 1.787223019 \| \| 1.785515035 \| \| 1.782414971 \| \| 1.776845033 \| \| 1.776042058 \| \| 1.771767126 \| \| 1.759558961 \| \| 1.751492144 \| \| 1.747216195 \| \| 1.733358725 \| \| 1.732565357 \| \| 1.729650663 \| \| 1.727621282 \| \| 1.724379134 \| \| 1.7219341 \| \| 1.718530459 \| \| 1.712201872 \| \| 1.709800412 \| \| 1.707636469 \| \| 1.704033892 \| \| 1.682663508 \| \| 1.659358181 \| \| 1.658197864 \| \| 1.6579008 \| \| 1.656654268 \| \| 1.653406143 \| \| 1.651893641 \| \| 1.651488167 \| \| 1.648722724 \| \| 1.635442446 \| \| 1.633795172 \| \| 1.632488785 \| \| 1.631657058 \| \| 1.630968002 \| \| 1.627835895 \| \| 1.614789041 \| \| 1.614388881 \| \| 1.609904776 \| \| 1.596397647 \| \| 1.59189194 \| \| 1.589710174 \| \| 1.589496543 \| \| 1.589050166 \| \| 1.58440526 \| \| 1.571622508 \| \| 1.571077483 \| \| 1.570152811 \| \| 1.569990903 \| \| 1.568009615 \| \| 1.560415784 \| \| 1.560202913 \| \| 1.559728947 \| \| 1.558653063 \| \| 1.558557485 \| \| 1.557376532 \| \| 1.543990638 \| \| 1.543580613 \| \| 1.543542011 \| \| 1.540314053 \| \| 1.53891814 \| \| 1.536202186 \| \| 1.533775272 \| \| 1.531402401 \| \| 1.526745229 \| \| 1.518408482 \| \| 1.518130511 \| \| 1.517399725 \| \| 1.51460551 \| \| 1.509371096 \| \| 1.509330646 \| \| 1.504104276 \| \| 1.49525919 \| \| 1.492266694 \| \| 1.491421789 \| \| 1.490507912 \| \| 1.488121659 \| \| 1.483254672 \| \| 1.47577936 \| \| 1.47483962 \| \| 1.473308848 \| \| 1.4695444 \| \| 1.461721702 \| \| 1.460771517 \| \| 1.459497651 \| \| 1.457670719 \| \| 1.453321328 \| \| 1.445259938 \| \| 1.444634925 \| \| 1.436121722 \| \| 1.435039604 \| \| 1.432566158 \| \| 1.432216156 \| \| 1.431802355 \| \| 1.429333467 \| \| 1.426445377 \| \| 1.426354149 \| \| 1.422354756 \| \| 1.417304715 \| \| 1.417148716 \| \| 1.416139698 \| \| 1.413281076 \| \| 1.413163633 \| \| 1.410499978 \| \| 1.408192012 \| \| 1.407773182 \| \| 1.401829544 \| \| 1.394256895 \| \| 1.393866045 \| \| 1.389760054 \| \| 1.383697387 \| \| 1.381898729 \| \| 1.374918306 \| \| 1.37404262 \| \| 1.373174305 \| \| 1.36663386 \| \| 1.36254121 \| \| 1.35938373 \| \| 1.357633143 \| \| 1.346792647 \| \| 1.339230376 \| \| 1.337438052 \| \| 1.336912559 \| \| 1.33587283 \| \| 1.329997624 \| \| 1.329354947 \| \| 1.319686231 \| \| 1.30945342 \| \| 1.30942459 \| \| 1.307626111 \| \| 1.306570071 \| \| 1.306291218 \| \| 1.303198781 \| \| 1.29559576 \| \| 1.277650934 \| \| 1.273290661 \| \| 1.266830891 \| \| 1.26073686 \| \| 1.25988281 \| \| 1.253998083 \| \| 1.244998421 \| \| 1.231193541 \| \| 1.221469201 \| \| 1.218607817 \| \| 1.214251557 \| \| 1.17149863 \| \| 1.160404283 \| |
