# Supplementary material for: Fatty acid synthase inhibition improves hypertension-induced erectile dysfunction by suppressing oxidative stress and NLRP3 inflammasome-dependent pyroptosis through activating the Nrf2/HO-1 pathway
Source: Front Immunol. 2025 Jan 14;15:1532021. doi: 10.3389/fimmu.2024.1532021 (PMC11772187; doi:10.3389/fimmu.2024.1532021)
Supplement: Supplementary file 9 [file Table4.docx]

**Table S4. Differential expressed NEG metabolites between the normal and the spontaneously hypertensive rats (SHR) groups.**

| **Compound_ID** | **Name** | **P value** | **Log_2_FC** | **Vip** |
| --- | --- | --- | --- | --- |
| \| Com_1525_neg \| \| --- \| \| Com_302_neg \| \| Com_3854_neg \| \| Com_1361_neg \| \| Com_2640_neg \| \| Com_5173_neg \| \| Com_3303_neg \| \| Com_2033_neg \| \| Com_1961_neg \| \| Com_7205_neg \| \| Com_7269_neg \| \| Com_2213_neg \| \| Com_6080_neg \| \| Com_3923_neg \| \| Com_1260_neg \| \| Com_2692_neg \| \| Com_77_neg \| \| Com_3547_neg \| \| Com_854_neg \| \| Com_7239_neg \| \| Com_295_neg \| \| Com_4233_neg \| \| Com_4846_neg \| \| Com_2064_neg \| \| Com_1405_neg \| \| Com_3345_neg \| \| Com_676_neg \| \| Com_3252_neg \| \| Com_3334_neg \| \| Com_2322_neg \| \| Com_5271_neg \| \| Com_4904_neg \| \| Com_3550_neg \| \| Com_4418_neg \| \| Com_3082_neg \| \| Com_2609_neg \| \| Com_7545_neg \| \| Com_3172_neg \| \| Com_284_neg \| \| Com_4524_neg \| \| Com_720_neg \| \| Com_7051_neg \| \| Com_2281_neg \| \| Com_732_neg \| \| Com_488_neg \| \| Com_3054_neg \| \| Com_6919_neg \| \| Com_5982_neg \| \| Com_42_neg \| \| Com_1299_neg \| \| Com_797_neg \| \| Com_7099_neg \| \| Com_1891_neg \| \| Com_692_neg \| \| Com_4518_neg \| \| Com_59_neg \| \| Com_1819_neg \| \| Com_474_neg \| \| Com_814_neg \| \| Com_110_neg \| \| Com_629_neg \| \| Com_1463_neg \| \| Com_1083_neg \| \| Com_590_neg \| \| Com_772_neg \| \| Com_106_neg \| \| Com_375_neg \| \| Com_594_neg \| \| Com_5957_neg \| \| Com_2669_neg \| \| Com_6087_neg \| \| Com_5321_neg \| \| Com_2564_neg \| \| Com_56_neg \| \| Com_3836_neg \| \| Com_1224_neg \| \| Com_2605_neg \| \| Com_4_neg \| \| Com_4246_neg \| \| Com_1715_neg \| \| Com_52_neg \| \| Com_623_neg \| \| Com_2838_neg \| \| Com_6918_neg \| \| Com_831_neg \| \| Com_263_neg \| \| Com_2658_neg \| \| Com_1203_neg \| \| Com_270_neg \| \| Com_1336_neg \| \| Com_6170_neg \| \| Com_1294_neg \| \| Com_309_neg \| \| Com_868_neg \| \| Com_673_neg \| \| Com_779_neg \| \| Com_767_neg \| \| Com_4215_neg \| \| Com_7233_neg \| \| Com_96_neg \| \| Com_2219_neg \| \| Com_1905_neg \| \| Com_4590_neg \| \| Com_36_neg \| \| Com_2569_neg \| \| Com_6333_neg \| \| Com_5064_neg \| \| Com_1977_neg \| \| Com_4349_neg \| \| Com_6_neg \| \| Com_2492_neg \| \| Com_7089_neg \| \| Com_3564_neg \| \| Com_392_neg \| \| Com_3232_neg \| \| Com_4759_neg \| \| Com_3428_neg \| \| Com_103_neg \| \| Com_1433_neg \| \| Com_2632_neg \| \| Com_81_neg \| \| Com_2471_neg \| \| Com_3611_neg \| \| Com_282_neg \| \| Com_1728_neg \| \| Com_1858_neg \| \| Com_7_neg \| \| Com_2447_neg \| \| Com_3321_neg \| \| Com_986_neg \| \| Com_150_neg \| \| Com_5632_neg \| \| Com_3957_neg \| \| Com_7475_neg \| \| Com_2275_neg \| \| Com_471_neg \| \| Com_744_neg \| \| Com_2482_neg \| \| Com_161_neg \| \| Com_144_neg \| \| Com_1316_neg \| \| Com_1915_neg \| \| Com_5960_neg \| \| Com_5337_neg \| \| Com_3804_neg \| \| Com_3329_neg \| \| Com_297_neg \| \| Com_837_neg \| \| Com_3768_neg \| \| Com_6658_neg \| \| Com_4457_neg \| \| Com_3224_neg \| \| Com_1658_neg \| \| Com_1407_neg \| \| Com_765_neg \| \| Com_943_neg \| \| Com_5346_neg \| \| Com_4280_neg \| \| Com_1879_neg \| \| Com_3824_neg \| \| Com_141_neg \| \| Com_677_neg \| \| Com_5182_neg \| \| Com_4378_neg \| \| Com_127_neg \| \| Com_6659_neg \| \| Com_4073_neg \| \| Com_7485_neg \| \| Com_5967_neg \| \| Com_5155_neg \| \| Com_4593_neg \| \| Com_3489_neg \| \| Com_2807_neg \| \| Com_1348_neg \| \| Com_7008_neg \| \| Com_6982_neg \| \| Com_2467_neg \| \| Com_3880_neg \| \| Com_1245_neg \| \| Com_6607_neg \| \| Com_193_neg \| \| Com_6010_neg \| \| Com_16_neg \| \| Com_750_neg \| \| Com_4872_neg \| \| Com_3487_neg \| \| Com_2394_neg \| | PE (3:0/16:4)  Erythronolactone  Astilbin  3-[3-(beta-D-Glucopyranosyloxy)-2-hydroxyphenyl]propanoic acid  PS (2:0/20:1)  Salvinorin A  FAHFA (18:0/20:2)  3-Hydroxydecanoic acid  α-Cyclodextrin  FAHFA (7:0/20:4)  Kaempferitrin  LPE 19:0  LPC 19:0  PE (2:0/20:1)  LPC 17:0  Dimetghyl 4-Hydroxyisophthalate  Stearic Acid  L-Methionine sulfone  Phenylacetaldehyde  PG (16:0/16:1)  Phosphocreatine  Ethyl myristate  PA (2:0/20:5)  LPC 20:2  N-Isovalerylglycine  Minocycline  15(S)-HpETE  3-Indoleacrylic acid  6-Hydroxycaproic acid  3-Hydroxysebacic acid  trans-Petroselinic Acid  Tyr-Tyr-Tyr  PE (2:0/20:0)  L-Palmitoylcarnitine  4-(octyloxy)benzoic acid  N1-(4-cyclohexylphenyl)-2-[(4-methylphenyl)thio]acetamide  Lysopa 18:0  2-Ethylhexanoic acid  methyl {[(2-oxo-2H-pyran-6-yl)carbonyl]amino}methanethioate  N4-Acetylcytidine  2-(acetylamino)-4-(methylthio)butanoic acid  N-Acetyl-L-tyrosine  AcylGlcADG (12:0-20:4-16:4)  1,3-dipyridin-3-ylpropane-1,3-dione  Cer-NS (d17:1/38:0)  Bialaphos  NSI-189  2-{1-[2-(4-benzhydrylpiperazino)-2-oxoethyl]cyclopentyl}acetic acid  methadone-d9  N-Acetyl-D-galactosamine 4-sulfate  4-Hydroxymandelonitrile  Norfludiazepam  Flavin adenine dinucleotide  Arginine  11-deoxy Corticosterone  LysoPE 18:0  N-acetyl-L-ornithine  Glycine anhydride  Uridine monophosphate (UMP)  UDP-N-acetylglucosamine  Uridine 5'-monophosphate  D-2-Aminoadipic acid  N-{5-[(dimethylamino)sulfonyl]-2-methylphenyl}cyclohexanecarboxamide  LPC 14:0  Cytidine-5'-monophosphate  LPC 20:4  Histidine  FAHFA (18:2/20:4)  Lysopc 16:1  2'-Deoxyuridine-5-monophosphate  1-(4-methylphenyl)-3-(2-pyridylthio)pyrrolidine-2,5-dione  PEtOH (12:0-18:2)  LPC 17:1  N-Acetyl-α-D-glucosamine 1-phosphate  Kojic acid  Adenosine 5'-monophosphate  3,5-Diiodotyrosine  Docosahexaenoic Acid  LPE 15:0  (±)19(20)-DiHDPA  Pyroglutamic acid  LPE 22:4  FAHFA (20:3/22:5)  LPS 20:0  LPS 20:3  LPE 18:2  HexCer-NS (d29:3/24:2)  17(S)-HpDHA  LPS 22:4  Guanosine monophosphate (GMP)  Prostaglandin A1 ethyl ester  FAHFA (20:4/22:5)  (±)9-HpODE  (±)9(10)-EpOME  FAHFA (18:1/20:3)  PE (20:4e/2:0)  LPS 22:6  FAHFA (22:5/18:2)  FAHFA (18:1/18:2)  LPE 16:0  DGDG (16:0/16:2)  FAHFA (22:4/18:0)  Phosphopyruvic acid  Maltotriose  Undecanedioic acid  MGDG (22:0/18:1)  LPE 20:1  LPA 8:0  Benzyl cinnamate  Linoleic Acid  Naringenin  LPG 20:5  FAHFA (20:4/18:1)  LPC 22:6  Dibutyl sebacate  11-Ketoetiocholanolone  LPE 18:3  LPE 18:1  LPE 20:3  FAHFA (22:5/20:4)  LPE 20:4  FAHFA (22:5/22:5)  Inosine-5'-monophosphate (IMP)  D-(+)-Glucose  2-Hydroxymyristic acid  12-oxo Phytodienoic Acid  Oleic Acid  LPC 20:3  LPC 22:5  LPE 22:5  δ-Ribono-1,4-lactone  Cannabidiolic acid  LPE 17:1  OxPE (18:0-20:3+4O(1Cyc))  LPC 20:5  Lysope 18:1  FAHFA (18:2/18:1)  FAHFA (22:6/18:3)  3-Phosphoglyceric acid  Phosphoenolpyruvic acid  LPS 16:0  Lauric acid  DGDG (18:4/20:4)  LPG 24:0  Tauroursodeoxycholic acid  PE (22:6e/2:0)  Myristic Acid  Taurochenodeoxycholic Acid (sodium salt)  LPE 14:0  FAHFA (18:1/22:4)  DGDG (18:1/18:3)  trans-10-Heptadecenoic Acid  cholesteryl sulfate  Thymidine 5'-monophosphate  4-amino-2-(4-chlorophenyl)-6-(methylthio)pyrimidine-5-carbonitrile  PC (18:5e/16:0)  SM (d14:1/23:0)  HexCer-NDS (d18:0/14:0)  N-benzyl-N-isopropyl-N'-[4-(trifluoromethoxy)phenyl]urea  FAHFA (18:3/16:2)  LPE 22:6  LPC 16:1  15-Deoxy-δ12,14 -Prostaglandin J2  Linolelaidic Acid (C18:2N6T)  (±)9(10)-DiHOME  1,7-Bis(4-hydroxyphenyl)-3,5-heptanediol  Dihydroroseoside  FAHFA (14:1/16:3)  Prostaglandin E1  LPE 20:5  PA (2:0/8:0)  PE (9:0/20:5)  Dehydroepiandrosterone  13-Hpotre(R)  DGDG (16:1/16:3)  OxPE (18:1-20:4+1O(1Cyc))  LPS 16:1  Prostaglandin E2  LPS 18:3  Monensin  LPE 16:1  Levosulpiride  Palmitoleic Acid  FAHFA (16:1/18:3)  19-Nortestosterone  Tetradecanedioic acid  MGDG (18:0/16:1) | 0.027763913  0.001976186  0.028626866  0.029804756  0.015734635  0.000133096  0.020673835  0.0063201  0.00075691  0.010975208  0.023486285  0.004540837  0.000687585  0.000132674  9.84E-05  0.018267377  0.009978531  0.01853895  0.01457539  0.005713503  0.005229347  0.016934332  0.015025303  0.000535664  0.032057046  0.000166357  0.044736459  0.028610005  0.021264703  0.012844067  0.036340754  0.012063979  0.030066412  0.024356657  0.000497682  0.0060819  0.025712495  0.038479469  0.029837564  0.024439475  0.021588462  0.00351484  0.002461599  0.000141647  0.004866018  0.008960367  0.00156579  2.55E-05  0.035726702  0.006645169  0.002450268  0.00015918  0.044168354  0.021346024  0.007697507  0.036234059  0.04938465  0.03726879  0.003985515  0.010045301  0.030922786  0.043200453  0.012119822  0.004090796  0.025722119  0.004571632  0.038077565  0.003866765  0.006547832  0.03210594  0.042237515  0.004272291  0.03550331  0.000753767  0.030451075  0.003958671  0.009230888  0.00249007  0.017003514  0.001337939  0.026502862  0.003287176  2.70E-05  0.012138634  0.008290588  0.012393454  0.038936422  0.007095525  0.039720741  0.049843992  0.004783693  0.005658523  0.000138926  0.006880454  0.001501192  0.00651521  0.000227877  0.002003117  0.002901216  0.006597435  0.001450295  0.002052565  0.017878292  0.002389084  0.000975078  0.004160045  0.003047253  0.044783304  0.006278944  1.15E-05  0.005395898  0.012959791  0.000459536  0.004889021  0.000328862  0.006045269  0.01783226  0.000134884  0.010899907  1.29E-05  0.000545616  0.000127128  0.001704533  0.019100191  0.001656277  0.021593269  1.79E-05  0.007420676  0.001750647  0.000828337  0.006310461  5.18E-05  4.34E-05  0.005777452  0.001727202  0.000355818  9.24E-06  0.000517373  0.008243655  0.008603669  0.005517987  0.000223747  0.000823046  7.68E-06  0.028323048  0.004988147  6.95E-07  0.035471136  0.000962903  9.31E-05  0.000900457  1.59E-05  0.045563454  0.002614436  0.008403474  4.86E-05  0.000770092  0.000550515  1.07E-06  8.45E-06  0.00149883  0.001102487  1.29E-05  8.73E-05  2.68E-05  1.99E-05  0.033271351  1.46E-06  0.00020949  0.000433192  1.22E-06  5.72E-07  4.87E-06  4.16E-06  0.001962044  0.00055519  0.000143075  0.003337653  7.60E-05  1.11E-06  0.000127515  4.37E-07  5.74E-07  2.85E-08  1.18E-06  4.07E-06  8.30E-06 | -3.000332619  -2.327908215  -2.245465519  -2.090828555  -1.954745065  -1.903031931  -1.83144478  -1.813910445  -1.813840836  -1.741910459  -1.648440175  -1.570170825  -1.488850089  -1.450114059  -1.396127185  -1.382786405  -1.272865009  -1.238965619  -1.182121633  -1.025751437  -1.025243125  -1.008575339  -0.970848737  -0.968806158  -0.949155216  -0.890298455  -0.875399588  -0.856633278  -0.819471385  -0.794309928  -0.774145126  -0.718250178  -0.706339802  -0.682783865  -0.675539822  -0.673415946  -0.663829211  -0.66333834  -0.662705176  -0.660834645  -0.659382133  -0.644558802  -0.628899764  -0.618130952  -0.609564667  -0.609562128  -0.571454476  -0.536842683  -0.484708327  -0.474423565  -0.45708861  -0.452936022  -0.341994781  -0.29920643  -0.267951719  -0.258560238  -0.198068192  0.331560768  0.364657176  0.366308172  0.367121344  0.379741125  0.384780193  0.396791045  0.418842825  0.44286743  0.470493162  0.471014801  0.504290977  0.506998313  0.508146995  0.517382195  0.527362013  0.527945349  0.533588155  0.57416278  0.579442713  0.583406322  0.584410856  0.613951068  0.620706838  0.623251445  0.629935162  0.659405038  0.672032169  0.704164272  0.713773112  0.720222558  0.756258193  0.760029013  0.765447158  0.779625593  0.823660028  0.824761621  0.826387199  0.861950305  0.866611645  0.871895184  0.872753743  0.874979712  0.885505787  0.885616907  0.89340219  0.896175793  0.905074233  0.906055406  0.914265011  0.920558648  0.948492775  0.955071403  0.959296187  0.963572175  0.991187819  0.99986937  1.029420157  1.035273839  1.087587903  1.089357543  1.104366043  1.12010952  1.146329471  1.177627591  1.202553683  1.255185462  1.260388262  1.271322083  1.290159851  1.345127353  1.38030083  1.385670927  1.38764045  1.416667273  1.457098326  1.476135585  1.47638915  1.479544395  1.488157774  1.529005529  1.559189633  1.566989185  1.569920945  1.577107392  1.61488861  1.636938947  1.671058411  1.681159598  1.692375758  1.708813238  1.773365522  1.773548014  1.786113219  1.797188331  1.802812255  1.85854974  1.871232941  2.005805257  2.091930309  2.132258997  2.222107803  2.234040717  2.386819975  2.402484548  2.409022221  2.44451945  2.607075371  2.623009594  2.630729564  2.65194877  2.653373651  2.773574394  2.855725826  2.871962945  2.910346759  2.959287518  2.967350456  2.985744583  3.067722366  3.140473818  3.214493684  3.352888619  3.423523162  3.579319249  3.700664164  3.708568169  3.8348713  3.835733107  4.440606355 | 1.164057621  1.475386214  1.209590402  1.186928073  1.246693978  1.586553664  1.191437845  1.359861097  1.494320271  1.309602037  1.193635143  1.361638632  1.504653337  1.597051696  1.585946208  1.234133674  1.287315448  1.218903083  1.270919325  1.369894388  1.354043071  1.250169381  1.304060786  1.549368464  1.183685691  1.584445387  1.107294837  1.185986771  1.210223946  1.278769946  1.148175377  1.272487175  1.149544152  1.17986926  1.561925756  1.347380262  1.162422752  1.099024558  1.182111342  1.176644649  1.244942322  1.434508329  1.438972619  1.581986924  1.36583403  1.340338009  1.46027686  1.627337018  1.103394722  1.375823878  1.489981444  1.577059684  1.072007669  1.171168089  1.297726463  1.097481353  1.14079033  1.136489628  1.426404587  1.337870114  1.182300381  1.174909386  1.307743699  1.429235745  1.22106815  1.375236269  1.113598926  1.456801424  1.387310994  1.222766715  1.126995946  1.395695155  1.193809435  1.544479283  1.180449603  1.408045762  1.348303104  1.472070264  1.315125194  1.487579588  1.183785946  1.411996255  1.639132289  1.349738524  1.383550419  1.287572555  1.145780709  1.378174583  1.171452335  1.125404853  1.435675437  1.388126044  1.598144005  1.382185257  1.500775355  1.402875216  1.574788879  1.483386105  1.448970624  1.386911353  1.518584222  1.473019639  1.243414903  1.424041244  1.508938541  1.450874338  1.459480629  1.092387705  1.337131426  1.664239275  1.348175778  1.247560735  1.580321975  1.387724535  1.594035804  1.337998333  1.228027226  1.616038881  1.309986338  1.661032307  1.523550807  1.621954502  1.466118213  1.221316175  1.472354085  1.255636385  1.664893526  1.359275888  1.485180021  1.486503608  1.353232184  1.616685442  1.643078238  1.410556621  1.458492562  1.570893388  1.663321815  1.553361346  1.326747509  1.325395856  1.389527608  1.587705974  1.49377467  1.667291519  1.179126774  1.407387609  1.700058739  1.146679567  1.544543821  1.601648447  1.529306299  1.660759756  1.13231514  1.455385189  1.304310892  1.62822273  1.516665887  1.523087071  1.696941551  1.663309174  1.475596748  1.517067566  1.645828974  1.618366994  1.638241353  1.652828114  1.179541513  1.691890378  1.582044327  1.549685939  1.689021574  1.698664192  1.675201513  1.673975915  1.482339674  1.560380685  1.616627916  1.430778684  1.626708777  1.688447236  1.612041733  1.699955248  1.698776824  1.703862422  1.691955081  1.68554795  1.652074331 |
