# Supplementary material for: Fatty acid synthase inhibition improves hypertension-induced erectile dysfunction by suppressing oxidative stress and NLRP3 inflammasome-dependent pyroptosis through activating the Nrf2/HO-1 pathway
Source: Front Immunol. 2025 Jan 14;15:1532021. doi: 10.3389/fimmu.2024.1532021 (PMC11772187; doi:10.3389/fimmu.2024.1532021)
Supplement: Supplementary file 10 [file Table5.docx]

**Table S5: Differential expressed genes between the normal and the spontaneously hypertensive rats (SHR) groups.**

| **ID** | **Log2FC** | **P-value** | **FDR** |
| --- | --- | --- | --- |
| ENSRNOG00000051885  ENSRNOG00000033564  ENSRNOG00000016456  ENSRNOG00000006025  ENSRNOG00000015538  ENSRNOG00000013408  ENSRNOG00000028993  ENSRNOG00000015086  ENSRNOG00000022239  ENSRNOG00000022932  ENSRNOG00000045797  ENSRNOG00000001821  ENSRNOG00000048472  ENSRNOG00000009686  ENSRNOG00000043451  ENSRNOG00000003972  ENSRNOG00000019718  ENSRNOG00000009563  ENSRNOG00000012660  ENSRNOG00000031955  ENSRNOG00000049351  ENSRNOG00000003221  ENSRNOG00000005806  ENSRNOG00000049388  ENSRNOG00000012404  ENSRNOG00000012181  ENSRNOG00000046858  ENSRNOG00000013128  ENSRNOG00000019050  ENSRNOG00000002323  ENSRNOG00000046216  ENSRNOG00000033915  ENSRNOG00000024115  ENSRNOG00000019412  ENSRNOG00000010999  ENSRNOG00000061733  ENSRNOG00000012083  ENSRNOG00000009153  ENSRNOG00000023109  ENSRNOG00000021217  ENSRNOG00000001469  ENSRNOG00000026646  ENSRNOG00000067962  ENSRNOG00000010805  ENSRNOG00000069830  ENSRNOG00000008716  ENSRNOG00000013552  ENSRNOG00000031997  ENSRNOG00000038999  ENSRNOG00000064907  ENSRNOG00000001963  ENSRNOG00000027295  ENSRNOG00000028310  ENSRNOG00000061320  ENSRNOG00000070043  ENSRNOG00000020702  ENSRNOG00000024899  ENSRNOG00000046890  ENSRNOG00000021524  ENSRNOG00000047023  ENSRNOG00000015859  ENSRNOG00000010938  ENSRNOG00000017878  ENSRNOG00000020923  ENSRNOG00000037080  ENSRNOG00000030285  ENSRNOG00000066573  ENSRNOG00000064527  ENSRNOG00000015948  ENSRNOG00000015518  ENSRNOG00000015902  ENSRNOG00000010079  ENSRNOG00000002385  ENSRNOG00000062245  ENSRNOG00000024221  ENSRNOG00000066894  ENSRNOG00000010655  ENSRNOG00000028616  ENSRNOG00000016281  ENSRNOG00000000165  ENSRNOG00000013023  ENSRNOG00000029339  ENSRNOG00000006787  ENSRNOG00000018494  ENSRNOG00000048088  ENSRNOG00000014964  ENSRNOG00000067240  ENSRNOG00000020659  ENSRNOG00000005546  ENSRNOG00000001435  ENSRNOG00000010633  ENSRNOG00000032596  ENSRNOG00000001452  ENSRNOG00000017976  ENSRNOG00000001001  ENSRNOG00000016311  ENSRNOG00000069480  ENSRNOG00000046700  ENSRNOG00000045683  ENSRNOG00000005792  ENSRNOG00000021207  ENSRNOG00000036682  ENSRNOG00000006388  ENSRNOG00000036693  ENSRNOG00000019612  ENSRNOG00000016573  ENSRNOG00000032461  ENSRNOG00000008481  ENSRNOG00000069673  ENSRNOG00000019430  ENSRNOG00000023151  ENSRNOG00000018413  ENSRNOG00000012543  ENSRNOG00000002653  ENSRNOG00000045636  ENSRNOG00000014336  ENSRNOG00000010325  ENSRNOG00000005457  ENSRNOG00000017646  ENSRNOG00000003840  ENSRNOG00000019780  ENSRNOG00000054286  ENSRNOG00000007378  ENSRNOG00000047028  ENSRNOG00000023337  ENSRNOG00000018798  ENSRNOG00000003669  ENSRNOG00000000463  ENSRNOG00000011723  ENSRNOG00000009079  ENSRNOG00000014090  ENSRNOG00000003442  ENSRNOG00000017579  ENSRNOG00000003927  ENSRNOG00000015438  ENSRNOG00000005659  ENSRNOG00000065120  ENSRNOG00000007546  ENSRNOG00000059445  ENSRNOG00000005008  ENSRNOG00000056457  ENSRNOG00000016552  ENSRNOG00000001060  ENSRNOG00000055829  ENSRNOG00000061579  ENSRNOG00000013971  ENSRNOG00000001726  ENSRNOG00000034052  ENSRNOG00000019019  ENSRNOG00000016623  ENSRNOG00000015156  ENSRNOG00000003800  ENSRNOG00000004921  ENSRNOG00000060021  ENSRNOG00000031607  ENSRNOG00000028137  ENSRNOG00000008422  ENSRNOG00000060614  ENSRNOG00000033748  ENSRNOG00000001959  ENSRNOG00000017676  ENSRNOG00000003217  ENSRNOG00000016826  ENSRNOG00000039079  ENSRNOG00000061813  ENSRNOG00000049614  ENSRNOG00000017259  ENSRNOG00000007923  ENSRNOG00000023086  ENSRNOG00000003348  ENSRNOG00000024923  ENSRNOG00000013057  ENSRNOG00000005082  ENSRNOG00000053047  ENSRNOG00000000967  ENSRNOG00000007078  ENSRNOG00000016085  ENSRNOG00000005573  ENSRNOG00000060486  ENSRNOG00000030776  ENSRNOG00000008839  ENSRNOG00000027894  ENSRNOG00000058006  ENSRNOG00000034013  ENSRNOG00000058068  ENSRNOG00000005286  ENSRNOG00000005291  ENSRNOG00000026861  ENSRNOG00000008001  ENSRNOG00000006776  ENSRNOG00000042634  ENSRNOG00000048949  ENSRNOG00000027245  ENSRNOG00000012995  ENSRNOG00000007755  ENSRNOG00000007743  ENSRNOG00000014966  ENSRNOG00000031536  ENSRNOG00000009734  ENSRNOG00000012442  ENSRNOG00000003597  ENSRNOG00000049880  ENSRNOG00000017716  ENSRNOG00000027869  ENSRNOG00000049422  ENSRNOG00000057794  ENSRNOG00000040242  ENSRNOG00000047506  ENSRNOG00000038001  ENSRNOG00000023657  ENSRNOG00000028636  ENSRNOG00000019372  ENSRNOG00000011659  ENSRNOG00000012674  ENSRNOG00000047314  ENSRNOG00000061429  ENSRNOG00000037753  ENSRNOG00000005365  ENSRNOG00000046171  ENSRNOG00000020829  ENSRNOG00000048411  ENSRNOG00000070375  ENSRNOG00000064392  ENSRNOG00000029191  ENSRNOG00000024650  ENSRNOG00000004554  ENSRNOG00000004828  ENSRNOG00000007290  ENSRNOG00000006033  ENSRNOG00000047581  ENSRNOG00000001425  ENSRNOG00000008933  ENSRNOG00000019582  ENSRNOG00000016872  ENSRNOG00000006116  ENSRNOG00000007284  ENSRNOG00000028344  ENSRNOG00000039560  ENSRNOG00000006231  ENSRNOG00000052764  ENSRNOG00000008323  ENSRNOG00000036828  ENSRNOG00000027433  ENSRNOG00000015423  ENSRNOG00000070637  ENSRNOG00000000521  ENSRNOG00000050426  ENSRNOG00000046834  ENSRNOG00000007335  ENSRNOG00000008310  ENSRNOG00000047605  ENSRNOG00000017820  ENSRNOG00000017619  ENSRNOG00000021243  ENSRNOG00000023116  ENSRNOG00000048039  ENSRNOG00000055608  ENSRNOG00000008000  ENSRNOG00000014448  ENSRNOG00000000451  ENSRNOG00000011775  ENSRNOG00000000158  ENSRNOG00000049075  ENSRNOG00000000433  ENSRNOG00000002470  ENSRNOG00000053811  ENSRNOG00000011446  ENSRNOG00000003486  ENSRNOG00000021691  ENSRNOG00000049033  ENSRNOG00000049994  ENSRNOG00000055499  ENSRNOG00000053804  ENSRNOG00000017611  ENSRNOG00000004589  ENSRNOG00000010635  ENSRNOG00000007102  ENSRNOG00000022268  ENSRNOG00000028996  ENSRNOG00000022429  ENSRNOG00000001843  ENSRNOG00000030462  ENSRNOG00000069004  ENSRNOG00000013223  ENSRNOG00000013975  ENSRNOG00000018755  ENSRNOG00000019728  ENSRNOG00000015675  ENSRNOG00000036865  ENSRNOG00000019751  ENSRNOG00000015416  ENSRNOG00000052157  ENSRNOG00000015727  ENSRNOG00000054203  ENSRNOG00000014039  ENSRNOG00000012982  ENSRNOG00000063284  ENSRNOG00000014603  ENSRNOG00000063618  ENSRNOG00000063216  ENSRNOG00000017448  ENSRNOG00000011228  ENSRNOG00000005053  ENSRNOG00000008609  ENSRNOG00000019189  ENSRNOG00000027767  ENSRNOG00000011151  ENSRNOG00000031211  ENSRNOG00000037563  ENSRNOG00000002911  ENSRNOG00000008050  ENSRNOG00000014683  ENSRNOG00000001338  ENSRNOG00000065011  ENSRNOG00000011668  ENSRNOG00000019138  ENSRNOG00000034139  ENSRNOG00000019745  ENSRNOG00000037446  ENSRNOG00000011360  ENSRNOG00000020546  ENSRNOG00000004201  ENSRNOG00000034191  ENSRNOG00000007805  ENSRNOG00000025994  ENSRNOG00000024382  ENSRNOG00000052070  ENSRNOG00000033658  ENSRNOG00000047349  ENSRNOG00000029662  ENSRNOG00000029071  ENSRNOG00000021663  ENSRNOG00000028945  ENSRNOG00000065935  ENSRNOG00000064007  ENSRNOG00000004487  ENSRNOG00000016325  ENSRNOG00000015149  ENSRNOG00000053449  ENSRNOG00000011989  ENSRNOG00000018615  ENSRNOG00000025415  ENSRNOG00000028047  ENSRNOG00000017672  ENSRNOG00000013736  ENSRNOG00000004708  ENSRNOG00000009826  ENSRNOG00000026143  ENSRNOG00000038132  ENSRNOG00000034229  ENSRNOG00000020991  ENSRNOG00000005278  ENSRNOG00000002158  ENSRNOG00000069600  ENSRNOG00000029195  ENSRNOG00000020276  ENSRNOG00000013306  ENSRNOG00000019615  ENSRNOG00000070613  ENSRNOG00000007516  ENSRNOG00000019914  ENSRNOG00000003336  ENSRNOG00000007139  ENSRNOG00000004744  ENSRNOG00000033528  ENSRNOG00000047124  ENSRNOG00000063127  ENSRNOG00000009334  ENSRNOG00000010263  ENSRNOG00000036571  ENSRNOG00000011039  ENSRNOG00000004084  ENSRNOG00000067479  ENSRNOG00000013097  ENSRNOG00000040122  ENSRNOG00000050123  ENSRNOG00000017178  ENSRNOG00000005542  ENSRNOG00000048686  ENSRNOG00000019390  ENSRNOG00000000122  ENSRNOG00000011445  ENSRNOG00000059753  ENSRNOG00000070083  ENSRNOG00000018436  ENSRNOG00000013970  ENSRNOG00000036669  ENSRNOG00000047198  ENSRNOG00000045654  ENSRNOG00000064680  ENSRNOG00000005574  ENSRNOG00000006709  ENSRNOG00000003384  ENSRNOG00000037206  ENSRNOG00000004441  ENSRNOG00000009468  ENSRNOG00000018867  ENSRNOG00000039865  ENSRNOG00000046460  ENSRNOG00000048275  ENSRNOG00000053026  ENSRNOG00000020837  ENSRNOG00000000156  ENSRNOG00000012318  ENSRNOG00000004302  ENSRNOG00000008040  ENSRNOG00000060237  ENSRNOG00000019093  ENSRNOG00000014075  ENSRNOG00000016681  ENSRNOG00000021560  ENSRNOG00000068526  ENSRNOG00000068378  ENSRNOG00000006857  ENSRNOG00000009339  ENSRNOG00000026053  ENSRNOG00000011107  ENSRNOG00000010478  ENSRNOG00000056493  ENSRNOG00000002364  ENSRNOG00000001859  ENSRNOG00000064052  ENSRNOG00000026548  ENSRNOG00000006236  ENSRNOG00000028711  ENSRNOG00000022067  ENSRNOG00000018656  ENSRNOG00000011332  ENSRNOG00000006620  ENSRNOG00000012972  ENSRNOG00000016838  ENSRNOG00000015654  ENSRNOG00000008412  ENSRNOG00000032150  ENSRNOG00000012609  ENSRNOG00000000632  ENSRNOG00000011654  ENSRNOG00000019068  ENSRNOG00000053272  ENSRNOG00000004197  ENSRNOG00000021763  ENSRNOG00000033335  ENSRNOG00000023130  ENSRNOG00000031849  ENSRNOG00000023412  ENSRNOG00000042326  ENSRNOG00000002208  ENSRNOG00000002566  ENSRNOG00000067711  ENSRNOG00000023431  ENSRNOG00000004857  ENSRNOG00000004405  ENSRNOG00000017786  ENSRNOG00000016769  ENSRNOG00000006921  ENSRNOG00000018929  ENSRNOG00000062297  ENSRNOG00000068777  ENSRNOG00000019627  ENSRNOG00000063716  ENSRNOG00000032871  ENSRNOG00000011973  ENSRNOG00000026661  ENSRNOG00000066614  ENSRNOG00000007907  ENSRNOG00000005705  ENSRNOG00000001770  ENSRNOG00000062568  ENSRNOG00000008034  ENSRNOG00000031397  ENSRNOG00000006996  ENSRNOG00000007906  ENSRNOG00000014840  ENSRNOG00000019937  ENSRNOG00000013262  ENSRNOG00000018570  ENSRNOG00000003832  ENSRNOG00000019466  ENSRNOG00000013727  ENSRNOG00000070149  ENSRNOG00000002031  ENSRNOG00000010880  ENSRNOG00000061153  ENSRNOG00000011000  ENSRNOG00000070559  ENSRNOG00000036827  ENSRNOG00000001389  ENSRNOG00000018815  ENSRNOG00000018870  ENSRNOG00000038370  ENSRNOG00000022921  ENSRNOG00000000500  ENSRNOG00000029386  ENSRNOG00000002302  ENSRNOG00000021200  ENSRNOG00000013738  ENSRNOG00000023214  ENSRNOG00000002852  ENSRNOG00000002876  ENSRNOG00000005115  ENSRNOG00000018851  ENSRNOG00000036703  ENSRNOG00000030848  ENSRNOG00000052080  ENSRNOG00000056069  ENSRNOG00000018445  ENSRNOG00000068418  ENSRNOG00000010721  ENSRNOG00000007461  ENSRNOG00000015774  ENSRNOG00000048881  ENSRNOG00000026745  ENSRNOG00000006930  ENSRNOG00000025823  ENSRNOG00000017412  ENSRNOG00000002711  ENSRNOG00000020557  ENSRNOG00000022764  ENSRNOG00000002408  ENSRNOG00000006802  ENSRNOG00000001296  ENSRNOG00000016603  ENSRNOG00000015921  ENSRNOG00000003566  ENSRNOG00000060100  ENSRNOG00000014786  ENSRNOG00000014641  ENSRNOG00000019208  ENSRNOG00000006611  ENSRNOG00000005723  ENSRNOG00000009715  ENSRNOG00000001809  ENSRNOG00000017645  ENSRNOG00000006731  ENSRNOG00000001369  ENSRNOG00000005569  ENSRNOG00000011748  ENSRNOG00000008837  ENSRNOG00000014702  ENSRNOG00000047186  ENSRNOG00000012303  ENSRNOG00000014909  ENSRNOG00000011649  ENSRNOG00000050706  ENSRNOG00000013376  ENSRNOG00000008536  ENSRNOG00000028415  ENSRNOG00000004668  ENSRNOG00000010438  ENSRNOG00000005003  ENSRNOG00000003585  ENSRNOG00000011912  ENSRNOG00000065770  ENSRNOG00000060401  ENSRNOG00000012989  ENSRNOG00000001242  ENSRNOG00000018070  ENSRNOG00000036692  ENSRNOG00000028541  ENSRNOG00000048961  ENSRNOG00000018358  ENSRNOG00000045560  ENSRNOG00000046763  ENSRNOG00000049695  ENSRNOG00000029308  ENSRNOG00000014907  ENSRNOG00000010519  ENSRNOG00000002783  ENSRNOG00000015529  ENSRNOG00000009788  ENSRNOG00000011460  ENSRNOG00000025634  ENSRNOG00000037302  ENSRNOG00000006653  ENSRNOG00000012835  ENSRNOG00000005378  ENSRNOG00000010045  ENSRNOG00000030143  ENSRNOG00000014143  ENSRNOG00000029670  ENSRNOG00000029055  ENSRNOG00000008098  ENSRNOG00000047741  ENSRNOG00000012719  ENSRNOG00000008986  ENSRNOG00000062737  ENSRNOG00000063044 | -6.364780895  4.638900632  -3.689182119  5.654016817  5.427103287  -2.718159469  -12.44173582  6.69970097  3.45417484  -2.586122573  7.429866991  7.401292277  -1.537057792  6.80713805  -1.969099524  4.798334917  -1.936250761  -3.430444358  2.357670369  6.396250996  4.124514188  -1.459642925  8.026523443  5.310415031  8.272034326  4.999868775  3.59479077  4.656581466  2.399781002  -2.424012017  -2.484818353  3.863015913  5.885741896  6.956158321  1.781946514  -1.659795994  -1.970978406  7.569256621  -1.69632361  -1.500876392  1.382118007  -2.073606459  -2.472804743  5.846815858  -2.504066906  5.74176658  8.350447066  -1.366383202  1.874885288  -13.688651  2.335370842  -1.260190975  2.916091112  -1.661050827  3.629884124  1.366942155  -2.067825146  -2.460056027  4.322377743  5.097913757  -2.195983418  5.304603125  7.36018867  1.727694976  1.994735837  1.716554684  -1.486052628  -4.273426208  1.607833032  1.923300949  -1.121749201  7.210745205  1.154702882  1.732759264  -11.46505617  -1.537399643  -5.618258911  7.899090309  1.058413174  4.658441113  -1.008132822  1.381804196  1.90602616  -1.680627717  2.148374811  3.826847107  2.314885936  -1.195507106  -7.044220625  1.473026191  4.391504685  1.49743989  -1.239933962  1.495095785  6.753772749  10.65254625  1.324505669  -1.61420153  1.213477762  10.07770587  8.570488231  1.781777607  2.437580759  2.015934089  -10.99364606  3.846531125  7.834207664  -1.250034045  1.821259999  1.755830056  -1.289904996  1.202212555  1.650116217  -1.085605835  3.583129702  2.238547662  3.115070865  1.57179782  -1.268087909  1.46220483  8.153393694  2.111181571  3.257540273  -1.374097298  1.446307597  3.013523444  -1.047692621  1.231557416  -1.6513939  1.79176657  1.576565286  3.520179317  -1.011205621  -1.037289036  3.45671713  2.806383345  5.536441505  1.438249368  1.364392994  1.6858446  4.333589874  1.073046127  -1.009087581  10.79631005  -5.316337039  1.731528487  1.10803839  -1.141396713  11.44207955  -1.299524893  -5.742992572  -1.154896274  1.888135697  4.835744818  -2.147405382  1.673109069  9.151439431  1.042380261  -4.533442071  1.826215638  1.126893087  1.217796163  1.713385453  9.812177306  2.00581485  1.006170687  1.730279547  1.315272064  1.57039884  1.60492094  1.802814412  1.523224955  1.735871694  1.976825746  1.965328107  1.107449496  -1.575390906  -1.130846459  -4.070265762  -1.030689204  1.148994707  2.088507825  1.012706323  1.263355621  3.056786026  1.733424055  -1.402258118  2.400449069  -1.213785668  5.431552586  10.16406888  2.571165503  1.563210541  2.911317611  1.32119911  2.89576827  1.362356007  -1.726080398  -1.935909795  -1.392473776  1.826578008  1.906973293  3.75886461  -1.219835584  -1.113521676  1.186848813  1.625722923  -1.295089857  1.270346964  4.292852523  2.612739544  2.775953891  4.231775034  9.530731451  1.913928546  -1.088230274  -2.443163743  5.598108909  3.27147618  1.239724892  1.707962167  7.080100049  -2.846977694  1.214124805  1.575567024  -1.019583007  5.525800428  1.286081353  -1.014577524  2.476732255  1.814328844  1.504234349  1.266593113  -1.258394833  -1.552526371  -1.035823018  1.615111435  -1.137881125  -1.128963684  11.78163293  -1.561967903  1.815785037  1.569261389  1.769235864  1.23965605  -2.802362341  5.511752654  1.802904797  3.164165732  6.608069968  -1.164481204  -1.171086939  4.22881869  1.630686421  1.119471294  3.065163417  -5.993976212  -2.828076212  -2.320436463  -1.827016553  2.221175707  2.485366464  1.635424785  -1.020440651  1.019987279  -1.73010954  3.476321241  2.019604558  1.311322162  1.454159516  -1.966228486  2.261837645  -1.358603818  -10.59618976  1.32268284  -1.246258411  -1.057807223  5.191242255  -1.869364584  -1.433998475  -1.206887678  1.366838227  8.929751553  1.308691029  3.578842894  1.164254831  1.33136827  -1.792505915  -5.077064102  -1.627801772  2.116249022  1.557568734  -1.00014547  8.251087854  6.24024093  1.653837677  5.157676456  1.269825621  3.025943267  1.751265005  1.388111043  1.355025696  -1.078890051  4.153221072  1.004445099  1.262868111  1.126474745  8.379378367  1.088151314  14.14146856  6.479671039  1.341541543  9.320424504  -2.001402718  -1.997199654  1.385289762  -2.427773183  7.382408667  1.502456701  1.334362377  1.014072559  -1.001895676  1.059091049  2.462834203  -1.469302948  -1.853370834  -1.04141071  1.881355504  -9.508455064  1.383387428  1.574044756  -1.077633108  1.685311172  9.065191713  -1.148112923  2.129826303  8.727920455  2.968693421  -1.721347633  -3.042968187  3.03562391  -1.020199285  1.085482053  2.395820362  1.97573206  2.695784958  1.389993354  2.025314985  8.994353437  5.121533517  1.186943585  2.38466385  -4.217765543  1.168956099  1.277415717  5.728247786  -1.020823036  -1.266352108  -9.9825183  7.811214118  3.48906896  7.724387528  2.307971504  1.308111718  1.064866146  6.972318199  1.648690384  2.01720929  -2.170980606  8.460797161  -1.100556712  1.983873782  1.115303942  3.132940883  5.218773115  5.521372183  4.164571767  6.016808288  2.336892534  3.600492199  1.002724203  1.332499592  4.917307212  8.490293787  3.663772567  1.601406576  1.217444125  -1.069341031  3.3917921  2.412041247  1.009311343  5.610689326  -2.570483003  1.617884117  1.428386764  -1.804386714  4.298212026  8.567322342  -1.210049091  4.91064273  1.89954862  3.274638044  -1.108901517  1.548931854  -1.603172778  1.922147855  1.734432327  -1.378048391  1.323257155  3.365747123  1.851499846  -5.942514505  9.513727596  -1.091269032  1.335271769  -2.171162085  1.008918065  10.89658469  5.131037952  1.451588387  1.332033739  -1.090220307  2.44386623  1.700439718  1.039125956  1.155184256  7.933336576  1.812939555  2.810571635  1.311461096  -1.863736044  1.007114652  -1.744357883  1.105434439  2.948049374  1.193955626  1.67753984  -3.219282046  1.10885095  5.526650768  4.721898146  1.391839155  1.231239534  1.139718066  1.255934579  1.170175839  -1.279600874  -9.97441459  8.266786541  -1.241235028  1.326036284  5.226068079  5.260953491  1.055507889  1.04939997  1.908659688  -1.979535897  3.140673122  5.275414004  6.022085642  -2.79630743  2.833918527  1.607957623  2.69434596  -2.22370178  1.257085948  1.197939378  -1.091962228  2.009062185  -2.450899144  1.506089479  1.747740066  2.028289044  -1.091124115  4.858343706  1.224642414  1.582204001  1.926250606  1.491935936  1.422441535  2.263890859  2.449288304  7.874469118  -1.164284248  8.48112669  1.225841402  -1.066825609  1.795107862  -4.308671998  11.28809705  1.289115759  1.24023875  -1.279303445  1.836407886  6.026155705  1.027424809  1.158568211  1.700048706  1.009658155  2.068204564  -1.07472069  1.720152869  5.716207034  1.851008072  1.353215065  1.942438731  1.796771583  1.088197764  4.480206439  2.519488663  3.920117755  2.694173932  3.590456416  1.558074811  4.121015401  1.214498038  5.302421781  1.570246001  1.774933444  1.634231567  1.103953346  1.259839622  1.628534781  3.367636617  1.160048322  2.486656339  4.452422952  1.3101154  2.004865755  -1.250922757  1.097114676  10.27262978  6.811126531  1.924381678  1.60789363  1.361015825  1.100913351  -1.371613968  -1.769009847  4.28739682  4.424418029  -2.611556711  4.565775481  -1.066021518  1.102907552  8.226337509  1.143339639  1.193391661  1.544193569  -2.138116263  2.355558873  1.166444733  2.209770337  1.663759508  2.234797804  2.191242753  -1.272349939  2.563254372  5.191343985  1.068925349  1.067434502  1.265226119  2.985153612  7.191109793  1.171817061  1.67675305  -1.209018277  5.008988783  1.44499537  8.194756854  1.452468989  1.428843299  1.326441222  1.909156574  1.665774546  1.218310321  1.891000746  1.104621653  1.157779572  -1.173505526  2.020768865  -1.411522237  -1.23641748  -4.608809243  1.151852727  -8.51438531  3.478047297 | 3.36E-246  9.72E-54  2.07E-47  5.23E-35  1.72E-30  3.63E-29  1.38E-28  2.51E-28  8.71E-28  1.05E-27  5.76E-27  1.69E-23  4.61E-23  8.21E-23  1.04E-22  9.65E-22  3.22E-21  5.86E-20  1.20E-19  1.82E-19  6.12E-19  6.92E-19  3.74E-18  6.92E-18  8.84E-18  3.49E-17  3.84E-17  4.97E-17  8.39E-17  8.63E-17  8.88E-17  9.66E-17  1.54E-16  2.07E-16  2.38E-16  3.59E-16  4.26E-16  7.90E-16  8.48E-16  9.57E-16  1.13E-15  1.60E-15  4.74E-15  5.26E-15  6.70E-15  7.40E-15  1.17E-14  1.28E-14  1.41E-14  1.45E-14  1.61E-14  3.41E-14  6.18E-14  6.62E-14  1.18E-13  1.40E-13  1.99E-13  2.83E-13  3.07E-13  3.10E-13  3.67E-13  3.78E-13  4.77E-13  4.78E-13  7.76E-13  7.92E-13  1.13E-12  1.26E-12  1.34E-12  1.34E-12  1.94E-12  3.28E-12  4.04E-12  4.39E-12  5.03E-12  6.19E-12  1.03E-11  1.31E-11  1.72E-11  2.81E-11  2.89E-11  3.03E-11  3.22E-11  3.69E-11  4.56E-11  5.14E-11  6.57E-11  7.53E-11  8.47E-11  1.08E-10  1.09E-10  1.21E-10  1.36E-10  1.42E-10  1.74E-10  1.82E-10  2.08E-10  2.33E-10  2.62E-10  3.45E-10  3.95E-10  4.41E-10  6.16E-10  6.20E-10  6.54E-10  1.01E-09  1.24E-09  1.34E-09  1.36E-09  1.37E-09  1.66E-09  1.67E-09  1.80E-09  2.06E-09  2.08E-09  2.11E-09  2.18E-09  2.38E-09  2.44E-09  2.87E-09  3.01E-09  3.75E-09  4.60E-09  4.89E-09  5.60E-09  6.03E-09  6.11E-09  7.00E-09  8.90E-09  1.04E-08  1.05E-08  1.11E-08  1.13E-08  1.22E-08  1.31E-08  1.32E-08  1.50E-08  1.59E-08  1.60E-08  2.04E-08  2.28E-08  2.39E-08  2.56E-08  2.68E-08  2.94E-08  3.38E-08  3.41E-08  3.69E-08  3.96E-08  3.99E-08  4.52E-08  5.15E-08  5.52E-08  5.56E-08  5.93E-08  6.30E-08  6.52E-08  8.19E-08  8.26E-08  8.31E-08  8.32E-08  9.44E-08  9.65E-08  1.01E-07  1.04E-07  1.25E-07  1.27E-07  1.31E-07  1.35E-07  1.46E-07  1.52E-07  1.53E-07  1.61E-07  1.65E-07  1.74E-07  1.95E-07  2.02E-07  2.12E-07  2.19E-07  2.20E-07  2.32E-07  2.32E-07  2.34E-07  2.37E-07  2.43E-07  2.56E-07  3.08E-07  3.22E-07  3.62E-07  4.33E-07  4.35E-07  4.53E-07  4.97E-07  5.02E-07  5.24E-07  5.42E-07  5.83E-07  5.86E-07  6.48E-07  6.53E-07  6.80E-07  6.88E-07  7.49E-07  7.52E-07  7.94E-07  9.17E-07  1.13E-06  1.28E-06  1.36E-06  1.38E-06  1.54E-06  1.64E-06  1.76E-06  1.77E-06  1.78E-06  1.82E-06  1.83E-06  2.05E-06  2.19E-06  2.23E-06  2.33E-06  2.35E-06  2.42E-06  2.49E-06  2.70E-06  2.83E-06  2.83E-06  2.93E-06  3.12E-06  3.20E-06  3.36E-06  3.65E-06  3.76E-06  4.09E-06  4.12E-06  4.18E-06  4.20E-06  4.25E-06  4.46E-06  4.54E-06  4.62E-06  4.70E-06  5.06E-06  5.07E-06  5.08E-06  5.44E-06  6.04E-06  6.37E-06  6.40E-06  7.01E-06  7.56E-06  8.32E-06  8.40E-06  8.43E-06  8.48E-06  9.08E-06  1.00E-05  1.02E-05  1.12E-05  1.13E-05  1.15E-05  1.19E-05  1.31E-05  1.33E-05  1.36E-05  1.44E-05  1.49E-05  1.57E-05  1.60E-05  1.63E-05  1.65E-05  1.69E-05  1.77E-05  1.79E-05  1.86E-05  1.87E-05  1.90E-05  1.93E-05  1.98E-05  1.99E-05  2.09E-05  2.11E-05  2.21E-05  2.24E-05  2.39E-05  2.43E-05  2.45E-05  2.49E-05  2.53E-05  2.64E-05  2.67E-05  2.71E-05  2.74E-05  2.92E-05  2.96E-05  3.03E-05  3.12E-05  3.14E-05  3.44E-05  3.46E-05  3.53E-05  3.79E-05  3.80E-05  3.85E-05  4.05E-05  4.24E-05  4.38E-05  4.41E-05  4.68E-05  4.76E-05  4.78E-05  5.38E-05  5.45E-05  5.69E-05  5.70E-05  5.87E-05  5.93E-05  5.99E-05  6.05E-05  6.15E-05  6.27E-05  6.63E-05  6.64E-05  6.85E-05  7.00E-05  7.13E-05  7.15E-05  7.42E-05  7.63E-05  7.72E-05  7.81E-05  8.12E-05  8.20E-05  8.45E-05  8.67E-05  8.83E-05  8.83E-05  8.86E-05  9.12E-05  9.24E-05  9.57E-05  9.60E-05  9.71E-05  9.85E-05  9.97E-05  0.000100152  0.000106838  0.000107006  0.000109789  0.000116392  0.000119383  0.000146736  0.000147455  0.000150535  0.000150667  0.000152692  0.000161797  0.000162398  0.000163288  0.000176997  0.000187202  0.000188154  0.000190765  0.00019266  0.000193547  0.000195451  0.000196721  0.00019815  0.000202878  0.000205881  0.000207061  0.000210591  0.000211015  0.000212439  0.000212861  0.000213623  0.000215544  0.000218634  0.000219302  0.00022363  0.000224189  0.000225979  0.00022698  0.000228259  0.000229879  0.000229937  0.000236128  0.000238409  0.000245455  0.000246681  0.000247047  0.000249592  0.000268313  0.000270937  0.000275243  0.000281232  0.000282954  0.000283725  0.000290507  0.000296292  0.000298428  0.000298965  0.000299693  0.00030318  0.000308739  0.000309319  0.000311024  0.000314857  0.000323909  0.000324149  0.000333679  0.000334893  0.000345656  0.000347636  0.000349509  0.000357574  0.000357815  0.000371542  0.000394961  0.000395875  0.000401347  0.000407989  0.000408028  0.000418103  0.000430798  0.000431381  0.000449692  0.000462192  0.000465415  0.000468564  0.000480002  0.000494271  0.000512665  0.000518495  0.000518533  0.000526542  0.00052771  0.000534794  0.000545426  0.000561248  0.00056745  0.000578129  0.000596035  0.000600956  0.000601972  0.000623585  0.000624298  0.000642309  0.000647532  0.000671449  0.000672438  0.000680626  0.000717044  0.000717215  0.000721105  0.000727681  0.00073974  0.000742664  0.000750388  0.000771537  0.000777347  0.000788973  0.000796387  0.000803108  0.000810432  0.000815745  0.000819177  0.000819464  0.000825507  0.000852265  0.000876407  0.000882623  0.000889602  0.000917419  0.000917473  0.000919339  0.000947826  0.00095571  0.000961923  0.000966409  0.000972187  0.00097396  0.001010951  0.001038059  0.001044825  0.001050089  0.001052764  0.001053338  0.001061864  0.001084362  0.001090461  0.00110124  0.001103819  0.001108719  0.001117287  0.001121045  0.001135865  0.001147393  0.001165021  0.001192089  0.001196398  0.001234538  0.001260342  0.001269141  0.001279986  0.001299533  0.001334668  0.001366816  0.001387752  0.001400162  0.001418703  0.001445746  0.001454094  0.001458006  0.001474506  0.001486156  0.001501363  0.001517781  0.001537637  0.001549931  0.001559121  0.00157321  0.00158126  0.00158363  0.001586044  0.001609378  0.001659461  0.001674575  0.001678534  0.001732457  0.001753235  0.001802356  0.00181452  0.001851975  0.001859519  0.001869509  0.001899629  0.001944609  0.001952877  0.001980344  0.002041774  0.002056415  0.002099917  0.002251402  0.002259626  0.002274562  0.002284395  0.002289971  0.002295497  0.002318218  0.002375984  0.002378595  0.002431532  0.002436897  0.002473673  0.002483784  0.00253706  0.002588428  0.002611892  0.002669173  0.002699981  0.002732483  0.002737368  0.00275442  0.002754955  0.002768761  0.002820022  0.002862067  0.00289127  0.002926469  0.00306214  0.003085137  0.003103805  0.003126351  0.003141076  0.003259452  0.003342237  0.003374599  0.003401783  0.003406847  0.003412978  0.003419902  0.003455925  0.003469753  0.003476553  0.003485671  0.003490835 | 5.74E-242  8.30E-50  1.18E-43  2.24E-31  5.88E-27  1.03E-25  3.36E-25  5.36E-25  1.65E-24  1.80E-24  8.95E-24  2.41E-20  6.06E-20  1.00E-19  1.19E-19  1.03E-18  3.24E-18  5.57E-17  1.08E-16  1.55E-16  4.98E-16  5.38E-16  2.78E-15  4.93E-15  6.05E-15  2.21E-14  2.34E-14  2.93E-14  4.74E-14  4.74E-14  4.74E-14  5.00E-14  7.72E-14  1.01E-13  1.13E-13  1.66E-13  1.92E-13  3.46E-13  3.62E-13  3.99E-13  4.59E-13  6.35E-13  1.84E-12  2.00E-12  2.49E-12  2.69E-12  4.15E-12  4.45E-12  4.81E-12  4.84E-12  5.29E-12  1.10E-11  1.95E-11  2.06E-11  3.62E-11  4.21E-11  5.88E-11  8.21E-11  8.68E-11  8.68E-11  1.01E-10  1.03E-10  1.26E-10  1.26E-10  2.01E-10  2.02E-10  2.83E-10  3.13E-10  3.23E-10  3.23E-10  4.55E-10  7.57E-10  9.20E-10  9.88E-10  1.12E-09  1.36E-09  2.22E-09  2.81E-09  3.62E-09  5.86E-09  5.94E-09  6.16E-09  6.48E-09  7.34E-09  8.96E-09  9.99E-09  1.26E-08  1.43E-08  1.59E-08  1.99E-08  1.99E-08  2.17E-08  2.42E-08  2.50E-08  3.04E-08  3.13E-08  3.55E-08  3.94E-08  4.40E-08  5.72E-08  6.49E-08  7.17E-08  9.81E-08  9.81E-08  1.03E-07  1.56E-07  1.91E-07  2.04E-07  2.05E-07  2.05E-07  2.47E-07  2.47E-07  2.63E-07  2.98E-07  2.99E-07  3.01E-07  3.07E-07  3.33E-07  3.39E-07  3.93E-07  4.08E-07  5.04E-07  6.10E-07  6.43E-07  7.30E-07  7.81E-07  7.85E-07  8.93E-07  1.11E-06  1.28E-06  1.28E-06  1.35E-06  1.36E-06  1.46E-06  1.55E-06  1.55E-06  1.74E-06  1.83E-06  1.83E-06  2.29E-06  2.53E-06  2.64E-06  2.80E-06  2.92E-06  3.18E-06  3.63E-06  3.64E-06  3.92E-06  4.16E-06  4.16E-06  4.65E-06  5.27E-06  5.62E-06  5.62E-06  5.94E-06  6.22E-06  6.41E-06  7.95E-06  7.95E-06  7.95E-06  7.95E-06  8.92E-06  9.06E-06  9.41E-06  9.63E-06  1.15E-05  1.15E-05  1.18E-05  1.22E-05  1.31E-05  1.35E-05  1.35E-05  1.41E-05  1.44E-05  1.51E-05  1.69E-05  1.73E-05  1.81E-05  1.86E-05  1.86E-05  1.95E-05  1.95E-05  1.95E-05  1.97E-05  2.01E-05  2.07E-05  2.46E-05  2.56E-05  2.86E-05  3.38E-05  3.38E-05  3.51E-05  3.81E-05  3.83E-05  3.98E-05  4.10E-05  4.35E-05  4.35E-05  4.77E-05  4.79E-05  4.93E-05  4.96E-05  5.33E-05  5.33E-05  5.61E-05  6.45E-05  7.84E-05  8.79E-05  9.33E-05  9.37E-05  0.000104036  0.000110574  0.000118194  0.000118194  0.000118415  0.000120305  0.000120728  0.000134509  0.000141102  0.000143453  0.000148282  0.00014954  0.000152549  0.000156604  0.000168366  0.000175118  0.000175118  0.00018029  0.00018987  0.000193834  0.000201703  0.000215  0.00022063  0.000234764  0.000235406  0.000237994  0.000238332  0.000240342  0.000251488  0.000255511  0.000258122  0.000260722  0.000276426  0.000276426  0.000276426  0.000295002  0.000325509  0.000341248  0.00034171  0.000371044  0.000397662  0.000434983  0.00043779  0.000438099  0.000439017  0.000467439  0.000508258  0.000518053  0.000554537  0.000558256  0.000567419  0.000584901  0.000633378  0.000642049  0.000654363  0.000688029  0.000713616  0.000743426  0.00075566  0.000764859  0.000770579  0.000783019  0.000813097  0.000821552  0.000847936  0.00085178  0.000862136  0.000872193  0.000892204  0.000896111  0.000928227  0.000933325  0.000972356  0.000980227  0.00103944  0.001051632  0.001059285  0.00106981  0.001087513  0.001125966  0.001135418  0.001145576  0.001155057  0.001214323  0.001228074  0.0012464  0.001280585  0.001284605  0.001388743  0.00139497  0.001413676  0.001507979  0.001509419  0.001519333  0.001579958  0.001648098  0.001696233  0.001701491  0.001794006  0.001819701  0.001819701  0.002020573  0.002033385  0.002112587  0.002112587  0.002171438  0.002185533  0.002200489  0.002213734  0.002240833  0.002281329  0.002394263  0.002395102  0.002453226  0.002495806  0.002528389  0.002528389  0.002592624  0.002657406  0.002680848  0.002699403  0.002785457  0.002804493  0.002860247  0.002918323  0.002947986  0.002947986  0.00295062  0.003027133  0.003054915  0.003145999  0.00314998  0.00316816  0.003205621  0.003240647  0.003248012  0.00343767  0.00343767  0.003500757  0.003683796  0.003771502  0.004510539  0.004524501  0.004598314  0.004598314  0.004651805  0.004894293  0.004895129  0.004907367  0.005251829  0.005516313  0.005525328  0.005582819  0.005619037  0.005625705  0.005655145  0.005669748  0.005691724  0.00577898  0.005854757  0.005868791  0.005949096  0.005951254  0.005976163  0.005976163  0.005985306  0.006029231  0.006095702  0.00610437  0.006204655  0.006210059  0.006239421  0.006256962  0.006282095  0.00630408  0.00630408  0.006436465  0.006477977  0.006628376  0.006628376  0.006628376  0.006686174  0.007154037  0.007198074  0.007292175  0.00740606  0.00743994  0.007448767  0.007603442  0.007731191  0.007763208  0.007765365  0.007772455  0.007839103  0.007970783  0.007973701  0.008005588  0.008054495  0.008211996  0.008211996  0.008374313  0.008392455  0.008611677  0.008648405  0.008657188  0.00878895  0.00878895  0.009084429  0.009574859  0.009583437  0.009702149  0.009835826  0.009835826  0.01002215  0.010268422  0.010268422  0.010659756  0.010895617  0.010941405  0.010985222  0.011172065  0.011462136  0.011808564  0.011863793  0.011863793  0.012014865  0.012025456  0.012154482  0.012363239  0.012654725  0.012760913  0.012983963  0.013333563  0.013408538  0.013413685  0.013821083  0.013821083  0.014128308  0.014206638  0.014674852  0.0146777  0.014803106  0.015457664  0.015457664  0.015521923  0.01564376  0.015823406  0.015846283  0.015971208  0.016299552  0.016321423  0.016545197  0.016619107  0.016738924  0.016871011  0.016960945  0.01699692  0.01699692  0.017060155  0.017570649  0.017895219  0.017979633  0.018078695  0.018513033  0.018513033  0.018528801  0.019036539  0.019171408  0.019273411  0.019340634  0.019433511  0.01944621  0.02004427  0.02050836  0.020572693  0.020605127  0.020621533  0.020621533  0.020764667  0.021083999  0.021178488  0.021315158  0.021340923  0.021363146  0.021431604  0.021479567  0.021714835  0.021861868  0.022050245  0.022463049  0.022519428  0.023110062  0.023485411  0.023577048  0.023726947  0.024011153  0.02443247  0.024824923  0.025098494  0.025239826  0.025416196  0.025819485  0.02588741  0.025930056  0.026196233  0.026370627  0.026535471  0.026742684  0.02703679  0.027191733  0.027246354  0.027464486  0.027576845  0.027590023  0.027594114  0.027924759  0.028710653  0.028880081  0.028919178  0.029609429  0.029815456  0.03040875  0.030583784  0.031031476  0.031127361  0.031233418  0.031490354  0.031987796  0.032060004  0.032419595  0.03313957  0.03334553  0.033954289  0.036025433  0.036025433  0.036229772  0.03633187  0.036339739  0.036393636  0.036617984  0.037289207  0.037295936  0.037986583  0.038035625  0.038401542  0.038521196  0.039233634  0.03985307  0.04008251  0.040821975  0.041127783  0.041401484  0.041438762  0.041594473  0.041594473  0.041766014  0.042315183  0.042795794  0.043156935  0.043606173  0.045233386  0.04549446  0.04573028  0.04598319  0.046160045  0.047613069  0.048614612  0.048902186  0.049229359  0.049260931  0.049307861  0.049324514  0.049718196  0.049875147  0.049888977  0.049935962  0.049968059 |
