# Supplementary material for: Fatty acid synthase inhibition improves hypertension-induced erectile dysfunction by suppressing oxidative stress and NLRP3 inflammasome-dependent pyroptosis through activating the Nrf2/HO-1 pathway
Source: Front Immunol. 2025 Jan 14;15:1532021. doi: 10.3389/fimmu.2024.1532021 (PMC11772187; doi:10.3389/fimmu.2024.1532021)
Supplement: Supplementary file 11 [file DataSheet1.docx]

**Supplementary Methods:**

1. ***Metabolomics Analysis***

To extract metabolites, powdered tissue samples were mixed with a dilution solution (acetonitrile: methanol: water, 4: 4: 2) and incubated at -20°C for 60 minutes. Subsequently, the samples were centrifuged at 15,000 rpm, 4°C for 5 minutes. The supernatant was collected, dried using a vacuum freeze dryer, and redissolved in 100 μL of a mixed solution (acetonitrile: water, 1: 1). The samples were centrifuged again at 15,000 rpm, 4°C for 5 minutes, and the supernatants were analyzed by liquid chromatography electrospray ionization tandem mass spectrometry (LC-ESI-MS/MS).

The relative signal intensity of the metabolites was normalized using standard samples, and log2 transformation was applied to the relative intensity. Data were analyzed following quality control. Raw data were converted from MS raw data (.raw) to mzXML format using ProteoWizard. Orthogonal partial least squares discriminant analysis (OPLS-DA) was conducted using SIMCA-P 14.1 (Umetrics, Umea, Sweden). Variable importance projection (VIP) values were derived from the OPLS-DA. Differential accumulated metabolites (DAMs) were identified based on VIP > 1 and P value < 0.05. Metabolic pathway analysis was performed using the KEGG compound database ([http://www.example.com/kegg/compound/](http://www.example.com/kegg/compound/)).

1. ***Transcriptome Analysis***

The outer urethra membrane of the penile cavernous body in rats was removed, and tissue RNA was extracted using TRIzol reagent (Invitrogen, Waltham, MA, USA). The quality and integrity of the RNA were assessed using the RNA Nano 6000 assay kit on a Bioanalyzer 2100 system (Agilent Technologies, CA, USA). Subsequently, cDNA libraries were constructed using the TruSeq RNA Sample Preparation Kit (Illumina, San Diego, CA, United States) according to the manufacturer's recommendations and sequenced on an Illumina platform.

Differentially expressed genes (DEGs) were identified using the DESeq2 R package. Genes with a fold change (FC) > 1 and Q value < 0.05 were defined as DEGs. Gene Ontology (GO) , Kyoto Encyclopedia of Genes and Genomes (KEGG) ([https://www.genome.jp/kegg/](https://webvpn.njust.edu.cn/https/77726476706e69737468656265737421e7e056d22035665f730dc7a688/kegg/)) and Reactome (reactome.org/) analyses were performed using the clusterProfiler R package to investigate the functional enrichment of the DEGs. Protein-protein interaction (PPI) networks were generated using the Search Tool for Retrieving Interacting Genes (STRING, [https://string-db.org/](https://webvpn.njust.edu.cn/https/77726476706e69737468656265737421e3e35395293725547c4686be9f/)) and visualized with Cytoscape software ([https://cytoscape.org/](https://webvpn.njust.edu.cn/https/77726476706e69737468656265737421f3ee5593343369407b4686be9f/)). The top five genes in the PPI networks were identified using CytoHubba.
